# The Antarctic Krill *Euphausia superba* Shows Diurnal Cycles of Transcription under Natural Conditions

**DOI:** 10.1371/journal.pone.0068652

**Published:** 2013-07-17

**Authors:** Cristiano De Pittà, Alberto Biscontin, Alessandro Albiero, Gabriele Sales, Caterina Millino, Gabriella M. Mazzotta, Cristiano Bertolucci, Rodolfo Costa

**Affiliations:** 1 Dipartimento di Biologia, Università degli Studi di Padova, Padova, Italy; 2 Centro Ricerche Interdipartimentale Biotecnologie Innovative (C.R.I.B.I.), Università degli Studi di Padova, Padova, Italy; 3 BMR Genomics, Padova, Italy; 4 Dipartimento di Scienze della Vita e Biotecnologie, Università degli Studi di Ferrara, Ferrara, Italy; Karlsruhe Institute of Technology, Germany

## Abstract

**Background:**

Polar environments are characterized by extreme seasonal changes in day length, light intensity and spectrum, the extent of sea ice during the winter, and food availability. A key species of the Southern Ocean ecosystem, the Antarctic krill (*Euphausia superba*) has evolved rhythmic physiological and behavioral mechanisms to adapt to daily and seasonal changes. The molecular organization of the clockwork underlying these biological rhythms is, nevertheless, still only partially understood.

**Methodology/Principal Findings:**

The genome sequence of the Antarctic krill is not yet available. A normalized cDNA library was produced and pyrosequenced in the attempt to identify large numbers of transcripts. All available *E. superba* sequences were then assembled to create the most complete existing oligonucleotide microarray platform with a total of 32,217 probes. Gene expression signatures of specimens collected in the Ross Sea at five different time points over a 24-hour cycle were defined, and 1,308 genes differentially expressed were identified. Of the corresponding transcripts, 609 showed a significant sinusoidal expression pattern; about 40% of these exibithed a 24-hour periodicity while the other 60% was characterized by a shorter (about 12-hour) rhythm. We assigned the differentially expressed genes to functional categories and noticed that those concerning translation, proteolysis, energy and metabolic process, redox regulation, visual transduction and stress response, which are most likely related to daily environmental changes, were significantly enriched. Two transcripts of peroxiredoxin, thought to represent the ancestral timekeeping system that evolved about 2.5 billion years ago, were also identified as were two isoforms of the *EsRh1* opsin and two novel *arrestin1* sequences involved in the visual transduction cascade.

**Conclusions:**

Our work represents the first characterization of the krill diurnal transcriptome under natural conditions and provides a first insight into the genetic regulation of physiological changes, which occur around the clock during an Antarctic summer day.

## Introduction

The Antarctic krill (*Euphausia superba*, Dana 1982), a crustacean species with a circumpolar distribution [1], plays a key role in the Antarctic ecosystem as it is an important link between primary producers and organisms at high trophic levels [2]. The Antartic krill has adapted to almost all marine environments in the Southern Ocean, including abyssal depths [3] and the under-ice habitat [4]. Due to its high content in proteolytic enzymes and water-soluble proteins, it has a significant impact on aquaculture and the fishing industry [5]. Krill abundance has, nevertheless, declined by 80% over the past 30 years in concomitance with global temperature rises and the consequent decrease in the winter sea ice cover [6], a change which has had important implications not only with regard to krill stocks but also to food interactions in the Southern Ocean [7], [8] and to the fishing industry [9].

The polar pelagic environment is characterized by extreme seasonal changes causing considerable variations in day length, light intensity and spectrum, sea ice extent and food availability [4].

Variations in photoperiod and light intensity have important effects on physiological parameters in krill such as feeding and metabolic rate [10], [11]. In fact a transcriptome analysis of gene expression has uncovered considerable differences in summer *vs.* winter populations [12]. It has been suggested that krill has evolved an endogenous time-keeping mechanism that can perceive seasonal variations in light conditions. The photoperiodic response could be mediated by a system that use the tools of a circadian clock to sense changes in day length and therefore synchronize the seasonal rhythm of metabolic activity to the annual cycle of the photoperiod that varies from near-constant light in December to near-constant darkness in June [13], [14] *E. superba* exhibits a daily vertical migration pattern moving downward during the day and upward during the night within a 200 m water column [15], [16]. Vertical migration probably allows the crustacean to maximize food intake in the superficial waters during the night and to minimize predator risk by migrating to deeper waters during the day [17]–[19].

The molecular organization of the clockwork driving these biological rhythms is, nevertheless, still poorly understood in marine organisms in general and in particular in high latitude pelagic ones [20]. Only one cryptochrome gene (*Escry*), a cardinal component of the clockwork machinery, has been characterized in *E. superba* [21], a finding suggesting that a circadian clock might indeed exist in that crustacean such as already observed in other decapod species [13]. Subsequent long term laboratory experiments with live krill entrained under different light-dark regimes have confirmed that a circadian clock is at work and regulating key metabolic processes [14]. These results suggest that the circadian clock of the krill plays a role in controlling also seasonal events.

While there is considerable scientific knowledge about the krill's biology and ecology, the crustacean's genome sequence is not yet available and a systematic sequencing of cDNA libraries is an efficient approach for the identification of large numbers of transcripts [22], [23]. Clark and colleagues have recently generated the first 454 sequencing data and focused their attention on the typical stress related genes of the Heat Shock Protein family [24]. In order to increase the probability of identifying circadian clock genes, or clock-controlled genes, we developed and sequenced a normalized cDNA library from krill specimens sampled at different times of the day across a complete 24-hour cycle. Using 454 Titanium technology, we were able to identify 80,675 high-quality reads which were assembled into a total of 10,987 putative transcripts. The sequences generated by our group together with all those available from public databases were *de novo* assembled to create the first krill oligo DNA microarray platform, named Krill 1.1 (Agilent Technology), containing a total of 32,217 different probes. Gene expression signatures of the specimens collected in the Ross Sea were defined at five different time points. One thousand three hundred eight genes (8% of probes) differentially expressed during the 24-hour cycle were identified; 609 of the corresponding transcripts showed a significant sinusoidal expression pattern with about 40% of these showing a 24-hour periodicity.

The aim of this study was to identify the diurnal gene expression of the Antarctic krill and group differentially expressed genes into functional categories. This is the first study that characterizes the crustacean's transcriptome in its natural conditions and provides insight into the genetic regulation of physiological changes occurring around the clock on a typical summer day in the Antarctica. This work opens the door to future studies on the role of the krill's circadian clock during seasonal adaptation and in response to polar climate changes.

## Results and Discussion

### Next generation sequencing and reads assembly

In order to better characterize the krill's transcriptome and to increase the probability of identifying circadian clock or clock-controlled genes, total RNA was extracted from a variety of adult organs and tissues, including the head (the brain and the compound eyes), the abdomen, and the thoracopods, of specimens sampled at different times of the day over a 24-hour cycle. Equal amounts of RNA were mixed together to construct a normalized cDNA library which was pyrosequenced using Roche 454 GS FLX. The 454 reads produced were used for clustering and *de novo* assembly using Newbler 2.5.1. After eliminating adapter sequences and filtering out the low-quality reads, including too short ones (<20 nt) and repeats, a total of 80,675 (83.3%) high-quality reads were further processed. Clustering and assembly of these reads yielded 340 isotigs (58,036 reads) and 22,622 singletons. The isotigs had an average size of 407 bp; 9 isotigs were greater than 1Kb (Figure S1A). In the attempt to design the most comprehensive microarray platform of Antarctic krill, we assembled the good quality 454 reads generated by our group together with all the sequences available from public databases. Newbler 2.5.1, considered one of the best assembly programs in restoring full-length transcripts despite some degree of chimeric contigs, was used [25]. This assembling provided a total of 32,217 consensus sequences: 11,720 isotigs and 20,497 singletons longer than 300 bp as outlined in Table 1. Interestingly, despite the relatively small number of reads produced by our normalized library, the putative identification of new krill transcripts was increased by about 10% with respect to the massive sequencing of Clark's library [24]. About 15% of singletons (3,116 out of 20,497) were provided by our normalized library, confirming the validity of the normalization procedure adopted to increase the gene discovery rate. Assembled isotigs ranged in size from 300 bp to 8.558 bp, with an average size of 890 bp; 3,577 isotigs were larger than 1Kb. The average length of our assembled isotigs was longer than that previously reported for krill (average of 492 bp) [24] but similar to that found in other decapod crustaceans (*Macrobrachium rosenbergii* and *M. nipponense*) [26], [27], whose transcriptomes were likewise obtained using the Roche 454 GS FLX. In view of the availability of long expressed sequence tags (ESTs) previously produced by the Sanger method [22], [23], we were able to increase the length of putative transcripts facilitating read alignment during the *de novo* assembly and the annotation process that followed. As described in Figure S1B, a search was made for each non-redundant sequence in the NCBI nucleotide and UniProtUK databases using Blast-N and Blast-X with an e-value cut off of <e^−50^ and <e^−6^ respectively. Priority was given to the Blast-N hits, and alignments characterized by less than 30% of coverage were discarded. Finally, with regard to the five best hits, the software selected the description of the organism with the closest taxonomic distance to the krill. Overall, 35% of the consensus sequences (11,230 out of 32,217) were successfully annotated while the other 65% (20,987 out of 32,217) showed no or poor similarity matches and presumably representing completely unknown Antarctic krill transcripts. Although the percentage of annotated transcripts might appear rather low, the number of matches was higher compared to previous 454 sequencing of the krill (25%) [24] and other non-model marine invertebrates: 12% in *Mytilus galloprovincialis*, 17% in the Antarctic bivalve *Laternula elliptica*, 23.9% in larvae of the coral *Acropora millipora* and 24% in the Manila clam *Ruditapes philippinarum* [28]-[31].

**Table 1:** Assembly of all available *E. superba* sequences (454 reads and ESTs).

454 reads assembly	
454 trimmed reads	866,729
Assembled reads	619,668
Singletons	86,073
Repeats	1,653
Too short (<20 nt)	68,960

ESTs assembly	
Good quality ESTs available on public databases	6,170
Assembled ESTs	4,991
Singletons	416
Repeats	7
Outliers	215
Too short (<20 nt)	3

Global assembly of 454 reads and ESTs	
Total isotigs (≥ 300 bp)	11,720
Isotigs (≥ 1 Kb)	3,577
Mean isotig lenght (bp)	890
Total singletons (≥ 300 bp)	20,497
Consensus sequences (isotigs and singletons)	32,217

Trimmed reads: sequences processed for assembling; Assembled reads/ESTs: sequences assembled by using Newbler 2.5.1; Singletons: putative transcripts identified by one read/EST; Repeats: reads that multiple map in several contig; Outliers: problematic reads such as chimeric sequences; Too short (<20 nt): sequence shorter than 20 nt; Isotigs: putative transcripts identified by at least two reads; Consensus sequences: non-redundant sequences (singletons + isotigs).

### Design of *E. superba* microarray platform

One probe for each consensus sequence was designed to construct the high-density oligo DNA microarray. Probe design was carried out using the Agilent eArray interface in order to produce 60 mer oligo-probes. Microarrays were synthesized *in situ* using the Agilent ink-jet technology with 8 x 60K format. Each array included default positive and negative controls. Microarray custom platform, named “Krill 1.1” (eArray Design ID: 034463), showed 29,440 duplicate probes and 2,777 single probes (GEO Accession N.: GPL16269).

### Gene expression data analysis

**Figure 1:**
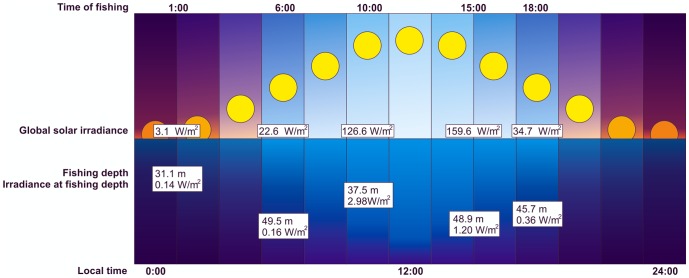
Environmental parameters of the Antarctic krill samples. Antarctic krill (adult specimens) were collected in the Ross Sea (longitude: 167°28′81′′ E – 179°54′68′′ W, latitude 68°40′54′′ S – 77°01′′81′′ S) in January 2004 during the XIX Italian Antarctic Expedition. The global solar irradiance was measured by a pyranometer (CM11, Kipp & Zonen; Delft, Netherlands) installed aboard the RV “Italica”. Time of fishing (local times: 01∶00, 06∶00, 10∶00, 15∶00, and 18∶00), global solar irradiance (W/m^2^), fishing depth (m), irradiance at fishing depth (W/m^2^) and local time (h) are reported. A blue color-coded scale provides a rough picture of the underwater irradiance at the fishing depth throughout the 24-hour cycle (actual values are also reported).

Gene expression profiling was performed using the “Krill 1.1” custom platform (Agilent) on specimens fished at different times during a 24-hour cycle (local times: 01∶00, 06∶00, 10∶00, 15∶00, and 18∶00) on a summer day/night in the Ross Sea region of Antartica (Figure 1) [21]. Total RNA was extracted from the head, including the brain and the compound eyes. In agreement with findings indicating that 9% to 30% of the transcriptome is clock-controlled in organisms such as cyanobacteria, *Arabidopsis*, *Drosophila* and mammals, a one-way ANOVA (Adjusted p-value ≤ 0.05, False Discovery Rate (FDR) ≤ 0.05) [32] identified 1,308 transcripts (8% of the probes) showing significantly different expression across the five different time points [33].

In order to specifically select genes also exhibiting a periodic oscillation, a time-domain approach was adopted using the CircWaveBatch V3.3 software (courtesy of Dr. Roelof Hut, http://www.euclock.org/). The main limit of our analysis was connected to the low number of time points (five) caused by the inaccessibility of extreme latitudes where it has been difficult to obtain more samples as usually required in circadian experiments. Despite the experimental and statistical limitations [34], [35] CircWaveBatch identified 609 (p<0.05) out of the 1,308 differentially expressed genes showing sinusoidal expression patterns (Figure S2A). Interestingly, we were able to confirm previous findings concerning daily fluctuations in abundance for *Escry* mRNA in the head (see Table S1), both in natural conditions [21] and in laboratory light-dark (LD) and constant dark (DD) regimes [14]. About 40% (238 out of 609) of periodically regulated genes showed only one peak of expression during the 24-hour cycle with a significant enrichment (47.8%) in the early day (01∶00 to 06∶00), while approximately 60% (371 out of 609) showed oscillatory patterns with a periodicity of approximately 12 hours, as recently reported for four key enzymes (citrate synthase, trypsin, aldo-keto reductase and β-N-acetylglucosamine) involved in carbohydrate metabolism [14]. The majority (56.5%) of the transcripts characterized by a 12 hour periodicity showed two high expression peaks at 10∶00±1 h and consequently at 22∶00±1 h, after the morning and evening light transitions represented, respectively, by the increase (10∶00, 2.98 W/m^2^) and the decrease (06∶00, 0.36 W/m^2^) of light irradiance at the fishing depth (Figure 1). These rhythms can be interpreted as bimodal patterns of a circadian output as already reported for *E. superba* [14].

Thirty-seven percent of the differentially expressed genes (490 out of 1,308) were successfully annotated while the remaining 63% transcripts (818 out of 1,308) showed no or poor similarity with publicly available sequences. A functional characterization of annotated transcripts was performed by DAVID (Table S1). As previously observed in fruit flies, mammals, and zebrafish, several biological processes influenced by daily environmental changes (transcription, translation, proteolysis, transport, energy and metabolic process, signal transduction, redox regulation, and stress response) are significantly represented (Figure S2B) [36]-[39]. More than 60% of the cycling transcripts, identified by CircWaveBatch and involved in transcription, proteolysis and cell growth, showed a bimodal expression with a 12-hour periodicity, while transcripts involved in protein synthesis were mainly characterized by a 24-hour one (Figure S2C).

### Peptide synthesis and processing

This is the most represented functional category with 47 differentially expressed annotated transcripts (Figure S2B), 32 of which showing a peak expression at 06∶00 h. This category also includes genes characterized by a sinusoidal expression pattern as in the case of those encoding ribosomal proteins (*60S ribosomal protein L34; ribosomal protein L3*), tRNA processing enzymes (*tRNA* (*guanine-N*(*7*)*-*)*-methyltransferase, tRNA 2-thiocytidine biosynthesis protein TtcA, glutamyl-prolyl-tRNA synthetase,* and *threonylcarbamoyladenosine tRNA methylthiotransferase CDKAL1*), and translation initiation factors (*eukaryotic translation initiation factor 3 subunit G, translation initiation factor eIF-2B subunit delta and translation initiation factor IF-3*). A diurnal oscillation in transcripts involved in protein synthesis rate was also paralleled by a concomitant up-regulation of those coding chaperones that accelerate and assist the correct folding process of nascent proteins (peptidyl-prolyl cis-trans isomerase 1, peptidyl-prolyl cis-trans isomerase FKBP14). A diurnal sinusoidal expression for genes encoding heat-shock protein 90 (hsp90) and hsp70 [40] was also observed. Not only protein synthesis but also protein degradation must be strictly regulated to maintain a diurnal oscillation of protein expression. Indeed previous studies demonstrated that proteolysis plays a key role in circadian clock entrainment [41] and in the regulation of critical clock-regulated pathways such as hormone signalling and light signal transduction [42]. One of the most relevant clock-controlled proteolytic pathways is represented by the specific ubiquitination of target proteins and their degradation in the proteasome. Transcripts for members of the ubiquitin conjugation cascade (*ubiquitin, two ubiquitin-conjugating enzyme E2, ubiquitin-protein ligase E3, ubiquitin conjugation factor E4*, and a *F-box containing protein*) as well as for 8 different structural and regulatory components of proteasome (*26S proteasome non-ATPase regulatory subunit 3, proteasome alpha 4 subunit, proteasome subunit beta type-5, proteasome zeta subunit, proteasome 25 kDa subunit, 26S proteasome non-ATPase regulatory subunit 5, proteasome subunit alpha type-2*, and *proteasome subunit beta type-2*) were differentially expressed in our dataset. Only those encoding ubiquitin, an ubiquitin-conjugating enzyme E2, and the proteasome 25 kDa subunit, showed a significant sinusoidal expression pattern after CircWaveBatch analysis.

### Transcription

This functional category (10.1%, Figure S2B) includes several oscillating transcripts encoding spliceosomal complex components (two different *serine/arginine-rich splicing factor 6*, and *U2 small nuclear ribonucleoprotein A*) involved in mRNA processing, some negative regulators of transcription (*transcriptional repressor NF-X1, transcriptional repressor rcnR*, and *transcriptional repressor p66-beta*), and epigenetic effectors. We identified two circadianly regulated chromatin- remodelling enzymes including peregrin, a component of the MOZ/MORF histone acetyltransferases complexes, and chromatin-remodeling complex ATPase chain isw-1, suggesting that there is an epigenetic modulation of circadian rhythmicity, as has been observed in other organisms [43]. Interestingly, as to be expected for genes involved in regulating a wide spectrum of physiological processes, the expression of genes belonging to this category is not confined to a specific temporal interval but is dispersed throughout the 24-hour cycle.

### Energetic and metabolic processes

**Figure 2:**
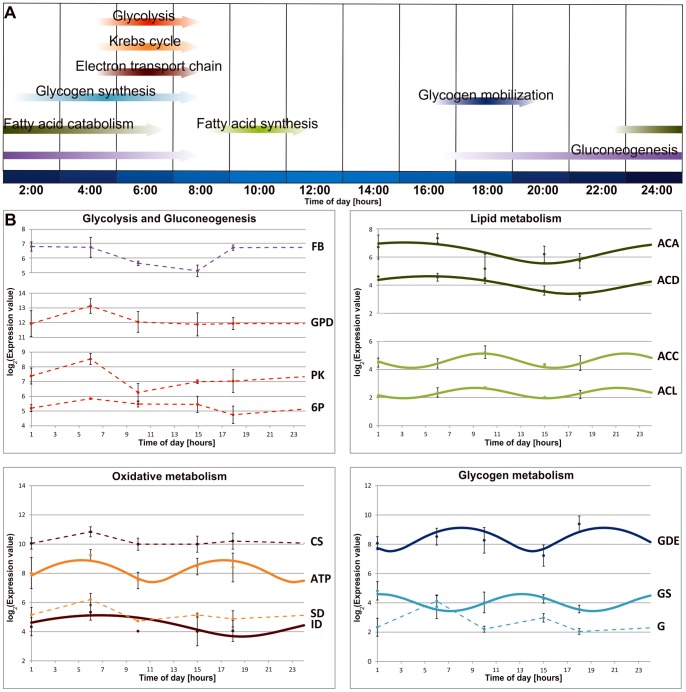
Oscillatory patterns of differentially expressed genes involved in energetic and metabolic processes. **A)** Schematic representation of the daily distribution of metabolic processes resulting from the transcriptional signature of several differentially expressed genes throughout the 24-hour cycle. The different metabolic processes are indicated by gradiently colored arrows showing the time of the day corresponding to the higher expression levels of gene groups. The lengths of the arrows and darker colors indicate, respectively, intervals and peaks of expression. Local times are indicated at the bottom of the figure where an indicative representation of light intensity is also shown. The breakdown of energy-yielding nutrients (glycolysis, the Krebs cycle and the electron transport chain) and energy storage pathways (glycogen synthesis and fatty acid synthesis) are specifically activated in the early morning, while glycogen mobilization, gluconeogenesis and fatty acids catabolism are used as a stored energy source in the evening and throughout the night. **B)** Gene expression profiles of transcripts involved in energetic and metabolic processes are represented. The color of each gene corresponds to the metabolic process in which it is involved. Dashed lines indicate differentially expressed genes identified by a one-way ANOVA test, while solid lines represent differentially expressed genes characterized by a sinusoidal expression patterns detected by CircWaveBatch analysis. Microarray expression values are represented as log_2_ mean ± standard deviation. Genes are represented with the following abbreviations: *fructose 1,6-bisphosphatase* (**FB**), *glyceraldehyde-3-phosphate dehydrogenase* (**GPD**), *pyruvate kinase* (**PK**), *6-phosphofructokinase* (**6P**), *citrate synthase* (**CS**), *ATP synthase b* (**ATP**), *succinate dehydrogenase type C* (**SD**), *isocitrate dehydrogenase [NAD] subunit beta mitochondrial* (**ID**), *Acetyl-CoA acetyltransferase* (**ACA**), *short-chain specific acyl-CoA dehydrogenase* (**ACD**), *Acetyl-CoA carboxylase* (**ACC**), *ATP citrate lyase subunit beta* (**ACL**), *glycogen debranching enzyme-like* (**GDE**), *glycogen synthase* (**GS**), and *Glycogenin-1* (**G**).

Forty-one differentially expressed genes were identified in this functional category and 30 of these had a peak of expression at 6∶00. These transcripts encode proteins involved in glycolysis and oxidative metabolism such as 6-phosphofructokinase catalyzing the rate-limiting step in glycolysis, nine components of ATP synthase and mitochondrial complexes involved in electron transport chain, and citrate synthase involved in the first step of the citric acid cycle. We observed a similar trend in expression with regard to the transcript encoding glicogenin, which acts as a primer for the polymeralization of glucose into glycogen. We detected an over-expression of the transcript for ATP citrate lyase and Acetyl-CoA carboxilase at 10∶00 h, probably related to the activation of the fatty acid and cholesterol biosynthetic pathways. Finally, genes involved in glycogen breakdown, gluconeogenesis, and fatty acid β-oxidation showed a peak of expression from late afternoon throughout the night. Among these we found glycogen debranching enzyme genes, which promote glycogen conversion to glucose, fructose 1,6-bisphosphatase, which catalyzes one of the last steps of gluconeogenesis, and acyl-CoA dehydrogenases, which catalyze the initial step of fatty acid β-oxidation in the mitochondria. Although a large majority of these genes did not show a significant sinusoidal oscillation when analyzed by CircWaveBatch, it is interesting to note that they showed peak expressions at specific times of the day, suggesting that they might play a relevant role in the temporal orchestration of metabolic processes (Figure 2). Transcripts for three key enzymes in fatty acid metabolism, (*ATP citrate lyase, Acetyl-CoA carboxylase* and *Acetyl-CoA Acetyl transferase*) showed, moreover, a significant sinusoidal oscillation.

Finally 29 out of 40 of these transcripts were involved in the glucose oxidative pathway, the major energy source in neurons. Our results are consistent with previous observations concerning murine suprachiasmatic nuclei (SCN) indicating that the circadian neuronal activity is related to oxidative metabolism rates [38]. A link between the endogenous circadian timing system and energetic catabolism has recently been desccribed in the Antarctic krill [14]. A circadian regulation of key metabolic processes has been implicated in anticipating environmental changes and interpreted as an adaptive energy management strategy [44], [45].

### Redox regulation

This functional category includes 12 genes. *Ferritin 1, Ferritin light chain*, and *heme binding protein 2* were characterized by a significant bimodal oscillation, with peaks at around 05∶00 and 17∶00. These results suggest that, just as has been recently reported in *Drosophila melanogaster,* iron metabolism could be modulated by a circadian clock in krill [46]. We also identified two krill cDNA sequences of *peroxiredoxin* characterized by identical coding sequences and different 5′ and 3′ Untranslated Regions (UTRs). These two peroxiredoxin transcripts are differentially expressed with a maximum at 06∶00 and a 24-hour periodicity. The amino acid sequence of krill peroxiredoxin showed more than a 75% similarity with peroxiredoxins previously found in other crustaceans such as the *Scylla paramamosain* and *Eriocheir sinensis* crabs.

**Figure 3:**
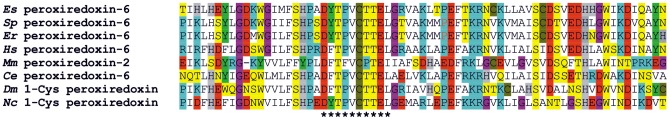
Multiple sequence alignment of the putative *E. superba* peroxiredoxin active site. Multiple sequence alignment of a protein region spanning 70 amino acids, including the peroxiredoxin active site (indicated by asterisks), had a high conservation level. Sequences are colored according to the residue type, and the alignment gap is indicated by dash (–). Hs: *Homo sapiens*, CAH59743; Mm: *Mus musculus*, AAA69475; Ce: *Caenorhabditis elegans*, NP_741287; Dm: *Drosophila melanogaster*, AAG47823; Nc: *Neurospora crassa*, XP_959621; Sp: *Scylla paramamosain*, ACJ53746; Er: *Eriocheir sinensis*, ACF35639.

A 70 amino acids domain containing the conserved catalytic site sequence of the *E. superba* peroxiredoxin is shown in Figure 3. Peroxiredoxins are thought to represent an ancestral timekeeping system, which developed approximately 2.5 billion years ago as a mechanism involved in reactive oxygen species (ROS) detoxification [47].

### Transport and signal transduction

Sixty-nine out of 336 genes are grouped into these two functional categories and are involved in vesicle trafficking, an essential mechanism in neuropeptide and neurotrasmitter processing and release. The gene encoding for the AP-1 complex subunit sigma-2 involved in protein sorting in trans-Golgi network showed a bimodal sinusoidal oscillation with peaks at 06∶00 and 18∶00. The gene for synaptoporin, an intrinsic membrane protein present in small synaptic vesicles, showed a sinusoidal expression pattern with a peak expression at 04∶00. Several oscillatory transcripts involved in the metabolism of glutamate, one of the major neurotransmitters, which is involved in photic entrainment in mammals, were also identified [48]. The transcript for the sodium dependent glutamate/aspartate transporter 2 (GLT1-like) is, for example, characterized by a 24-hour periodicity and its product plays a major role in the clearance of excitatory amino acid neurotransmitters in mammals [49]. We also found that genes for glutamine synthetase and glutamate dehydrogenase had, respectively, an expression peak at 24∶00 and 06∶00. The gene for GMP synthase/glutamine amidotransferase, a member of the amidohydrolase family, showed a sinusoidal expression pattern and a 12-hours periodicity. The encoded enzyme is also important for *de novo* synthesis of guanine nucleotides, involved (as cGMP) in visual phototransduction in invertebrates [50] as well as in photic entrainment in mammals [51]. The gene for the pigment dispersing factor (PDF) receptor is differentially expressed with a peak at 06∶00. PDF is a neuropeptide controlling circadian behavioral rhythms in Drosophila and in most artropods in which it is linked to an autocrine feedback signaling in clock neurons [52].

**Figure 4:**
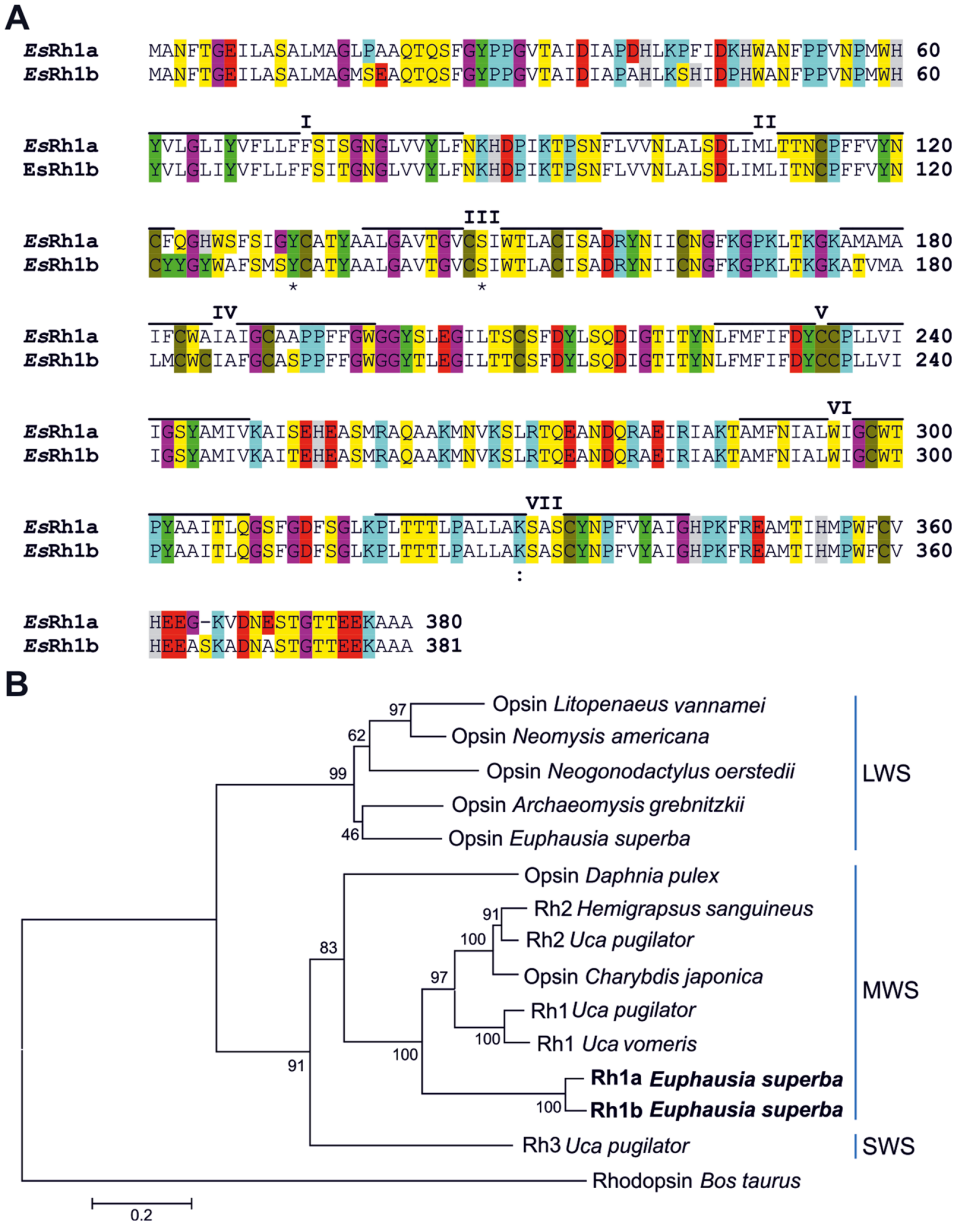
Phylogenetic relationships of ***E.superba*** **opsins.** **A)** **** Alignment of the amino acid sequences of *E.superba* Rh1a and Rh1b opsins obtained by ClustalW. Seven transmembrane domains were predicted using TMPred (http://www.ch.embnet.org/software/TMPRED_form.html), indicated by lines above the sequence and labelled with Roman numerals. Asterisks (*) indicate amino acids functionally related to the long/middle wavelength-sensitive opsins, a colon (:) indicates the attachment site for the retinal Shiff's base linkage to the retinal chromophore. Sequences are colored according to the residue type. An alignment gap is indicated by a dash (–). **B)** Phylogenetic relationships of the EsRh1 (Rh1a and Rh1b) and opsin orthologs were obtained by neighbor-joining analysis. Bootstrap confidence values are based on 1,000 replicates. Scale bars indicate amino acid substitutions per site. LWS, long-wavelength sensitive; MWS, middle-wavelength sensitive; SWS, short-wavelength sensitive. Accession numbers: *Litopenaeus vannamei*: ABH00987; *Neomysis Americana*: ABI48886; *Neogonodactylus oerstedii*: ACU00212; *Archaeomysis grebnitzkii*: ABI48867; *Euphausia superba* LWS: ABI48874; *Daphnia pulex*: EFX76309; *Uca pugilator* Rh2: ADQ01810; *Hemigrapsus sanguineus*: Q25158; *Charybdis japonica*: AEL33282; *Uca vomeris*: ACT31580; *Uca pugilator* Rh1: ADQ01809; *Uca pugilator* Rh3: E5G6F2; *Bos taurus*: NP001014890.

We selected from our dataset four differentially expressed genes linked to the visual system. Two transcripts were identified as encoding BcRh2 opsin like, a light-sensitive receptor involved in the visual transduction cascade in arthropods. These transcripts are full-length coding sequences. In view of the opsin structure, they showed a 92% similarity in their deduced amino acid sequences including, among others, the putative G-protein binding regions (QAKKMNV and DRY respectively at positions 261–267 and 157–159) and the lysine (K330) that is the site of attachment for the Shiff's base linkage to the retinal chromophore (Figure 4A). These novel *E. superba* opsins show specific amino acids (i.e. Y13 and S147) located in the third transmembrane domain that are typical of the long/middle wavelength-sensitive opsin [53]. These observations are supported by a phylogenetic analysis (Figure 4B) leading us to conclude that the novel krill opsins are more similar to Rh1 type opsin than to Rh2, as originally annotated. We decided consequently to re-name our opsins *EsRh1a* and *EsRh1b*. These genes show a peak expression at 06∶00. A diurnal opsin rhythm is considered a critical step in the pigment renewal process and its accumulation at light onset has already been described in the mouse [54], the rat [55], and the honey bee [56]. We also identified two sequences with a putative similarity to arrestin1 of *D. melanogaster and L. polyphemus*. These sequences showed a low level of homology and two different sinusoidal bimodal expression profiles with peaks, respectively, at 06∶00 and 15∶00 and 10∶00 and 18∶00. Diurnal fluctuations in *arrestin* mRNA levels have been already reported in different species, including two arthropods, the horseshoe crab [57] and the honey bee [56].

### Quantitative RT-PCR validation

Quantitative RT-PCR analysis was performed to quantify and validate the expression levels of some of the differentially expressed genes identified by microarray experiments. We selected nine genes (*26S proteasome non-ATPase regulatory subunit 3, arrestin 1, citrate synthase, heat shock cognate 70, heat shock protein 90-1, opsin EsRh1a, Peroxiredoxin 6, Phosphatidylinositide phosphatase SAC1, ubiquitin-conjugating enzyme E2*) characterized by expression peaks distributed throughout the 24-hour cycle. The housekeeping gene *18S rRNA* was used as an endogenous control. Despite the high level of inter-individual variability, the expression values obtained with qRT-PCR for the nine transcripts paralleled those obtained by microarray analysis (Figure S3).

## Conclusions

Massive sequencing of a normalized cDNA library has permitted us to increase the discovery of new transcripts of krill by about 10% with respect to what was available in databases of transcriptomes of this species. Bearing in mind the unduly large size (estimated to be 48 Gb) [58] of this species' genome sequence, this is an important accomplishment since it is not yet and probably will not be available in the near future. Using all available sequences, we created the first krill oligonucleotide microarray platform, which currently represents the most complete genomic tool to investigate its genomic expression. The first diurnal transcriptome characterization of krill was obtained in natural conditions on a summer day in the Antarctica. About 1,300 genes were found to be differentially expressed across the time points analysed and many of these showed a cyclic sinusoidal expression. Interestingly, genes belonging to a number of specific functional categories showed peak expressions limited to distinct temporal intervals, confining specific biological processes to particular times of the day. This chronological progression of biochemical and physiological events throughout the 24-hour cycle most likely reflects the workings of an endogenous circadian clock that controls krill metabolism. An internal timing mechanism that synchronises physiology and behaviour is important for the survival of an organism exposed to such dramatic seasonal changes and harsh environmental conditions. In the summer, a season characterized by favourable environmental conditions in terms of food availability and light intensity, the Antarctic krill has an active metabolism, allowing it to grow and to undergo the physiological modifications, such as body lipid accumulation, necessary to face unfavourable wintertime conditions characterized by low food supply. This work provides new insight concerning the *E. superba* circadian regulation in natural conditions and paves the way for a deeper understanding of its adaptive survival strategy to a harsh habitat and to fluctuations connected to global climate changes.

## Materials and Methods

### Ethics Statement

All animal work has been conducted according to relevant national and international guidelines. Krill fishings were conducted in accordance with the Convention for the Conservation of Antarctic Marine Living Resources (CCAMLR, 1980). Animals were collected during the XIX Italian Antarctic Expedition (2003–2004) and fishing permissions were obtained by the Italian Scientific Commission for Antartica (CSA) and Consortium of Programma Nazionale di Ricerche in Antartide (PNRA, Project N. 2003/1.3 “Molecular neurogenetics of circadian rhythmicity in *Euphausia superba*”).

### Specimen collection and total RNA isolation

Antarctic krill (*Euphausia superba*) were collected in the Ross Sea (longitude: 167°28′81′′ E – 179°54′68′′ W, latitude 68°40′54′′ S – 77°01′′81′′ S) in January 2004 during the XIX Italian Antarctic Expedition with procedures described in Mazzotta *et al*. (2010) [21]. Adult specimens were fished at different times throughout the 24-hour cycle (local times: 01∶00, 06∶00, 10∶00, 15∶00, and 18∶00) and stored at −40°C in RNA stabilization solution (RNA-later, Life Technologies). For each fishing, four specimens were dissected, and total RNA was extracted from the head (including the brain and the compound eyes), abdomen, and thoracopods, as described in De Pittà *et al*. (2008). All RNA samples were checked for quality by capillary electrophoresis (RNA 6000 Nano LabChip, Agilent Bioanalyzer 2100, Agilent Technologies). Equal amounts of total RNA (1 μg) derived from each tissue extracted and for each time point considered were pooled and the resulting total RNA pool was used to construct a normalized library.

### Normalized cDNA library construction and sequencing

We developed a new approach, specifically designed in the event of small amounts of starting material, by combining two different protocols: the “Whole Transcriptome Amplification (WTA)” [59], providing an accurate, rapid, uniform method of amplifying total RNA and “Duplex-Specific Nuclease (DSN) normalization” [60], [61] used to equilibrate the final representation of abundant and rare transcripts (Figure S4). Whole Transcriptome Amplification (WTA) was performed using the TransPlex WTA kit (Sigma-Aldrich) and following the manufacturer's protocol characterized by two steps; 1) library synthesis and 2) library amplification. 1) cDNA synthesis was performed using 150 ng of total RNA with non-self-complementary primers composed of a quasi-random 3′-end and a universal 5′-end. Overlapping 250–500 base fragments, flanked by a universal end sequence were randomly generated. 2) Universal-primer was used as forward and reverse primer to amplify cDNAs. The PCR reaction mixture was incubated for 16 cycles for 15 sec at 95°C (denaturation) and 5 min at 65°C (annealing and elongation). The PCR reaction was stopped after 16 cycles (exponential phase) in order to maintain differences in transcript expression. Excess of primers and nucleotides was removed using the GenElute PCR Clean-Up Kit (Sigma-Aldrich). The Trimmer-Direct cDNA normalization kit (Evrogen) was used to normalize the cDNA library. 1.5 μg of amplified cDNA was denatured at 95°C for 2 minutes and subsequently renaturated at 68°C for 5 hours. This procedure was carried out to equilibrate transcript expression levels of different abundance. The dsDNAs obtained after the renaturation process are, in fact, enriched in abundant transcripts, while less represented transcripts remain as single strand DNA. The dsDNAs were specifically degraded by the Red King crab nuclease (1 μl in a reaction volume of 20 μl for 25 minutes at 68°C). Afterwards the normalized ssDNA was amplified by a new 16 cycles PCR reaction using the same WTA universal primer. Normalized dsDNA was then diluted 1∶3, purified with the GenElute PCR Clean-Up Kit and the quantity/quality was respectively checked by fluorometric assay (Qubit, Life Technologies) and capillary electrophoresis (DNA 1000 LabChip, Agilent Technologies). The cDNA samples (300 ng), clonally amplified and bead-immobilized, were sequenced by the 454 GS FLX Titanium sequencer at BMR Genomics (Padova, Italy, www.bmr-genomics.it), using one lane of 70x75 picotiter plates, as described in the GS FLX Titanium Sequencing Method Manual (Roche). Bases were called with 454 software by processing the pyroluminescence intensity for each bead-containing well in each nucleotide incorporation. Raw data are accessible on the National Center for Biotechnology Information Short Read Archive (SRA Accession Number: SRS376031).

### 454 Assembly and Analysis

A total of 96,803 reads were produced from the normalized cDNA library and the adapter sequences were trimmed using the Univec database obtaining 80,675 good quality reads. In particular, good quality 454 reads generated by our group and all the available sequences of krill (ESTs and the 454 reads produced by Clark and colleagues [24]) were assembled into isotigs by using Newbler 2.5.1. Singletons were re-mapped on isotigs reducing the overlap to 20 bp in order to decrease the number of singletons (unique sequences), as described in Figure 1A.

### DNA microarray design

The global assembly of the 454 reads generated by our group and all the sequences available from public databases provided a total of 11,720 consensus sequences (isotigs) and 20,497 singletons longer than 300 bp. One probe for each consensus sequence was designed to construct a high-density oligo DNA microarray. Probes were designed using the Agilent eArray Custom Microarray Design Service (https://earray.chem.agilent.com/earray/index.jsp), which applies proprietary prediction algorithms to design 60 mer oligo-probes. Microarrays were synthesized *in situ* using the Agilent ink-jet technology with 8 x 60 K format. A total of 32,217 probes representing *E. superba* transcripts were successfully obtained. A custom microarray platform, named “Krill 1.1” (eArray Design ID: 034463), showed 29,440 duplicate probes and 2,777 single probes. Each array included default positive (1,011 probes) and negative (308 probes) controls. A correct match between each probe and its consensus sequence was verified using Blast-N. Only 1,623 out of 32,217 probes (5%) showed two or more hits with different consensus sequences and thus subject to cross-hybridization (false positives). These probes were excluded from the gene expression analysis that was carried out later. Probe sequences and other details on the microarray platform can be found in the Gene Expression Omnibus (GEO) database (http://www.ncbi.nlm.nih.gov/geo/) under accession number: GPL16269.

### Microarray labeling and hybridization

Gene expression profiling was carried out on krill specimens caught at different time points across the 24-hour cycle (local times: 1∶00, 6∶00, 10∶00, 15∶00, and 18∶00) using the Krill 1.1 custom platform (Agilent). RNA was obtained from the head of a single specimen at each time point. Four biological replicates were analyzed for a total of 20 microarray experiments. 800 ng of total RNA was labeled with “Agilent One-Color Microarray-Based Gene Expression protocol” according to the manufacturer's instructions. The synthesized cDNA was transcribed into cRNA and labeled with Cy3-dCTP. Labeled cRNA was purified with RNeasy Mini columns (Qiagen, Valecia, CA). The quality of each cRNA sample was verified by total yield and specificity calculated with NanoDrop ND-1000 spectrophotometer measurements (Nanodrop, Wilmington, DE). 1.65 μg of labeled cRNA was used in each reaction and hybridization was carried out at 65°C for 17 hours in a hybridization oven rotator (Agilent Technologies, Palo Alto, CA). The arrays were washed using Agilent Gene expression washing buffers and Stabilization and Drying Solution, as suggested by the supplier. Slides were scanned on an Agilent microarray scanner (model G2565CA) and Agilent Feature Extraction software version 10.5.1.1 was used for image analysis. Gene expression data are available in the GEO database with the accession number: GSE42292.

### Statistical analysis of gene expression data

Inter-array normalization of expression levels was performed with quantile [62] to correct possible experimental distortions. A normalization function was applied to the expression data of all the experiments and the values for within-arrays replicate spots were then averaged. Feature Extraction Software, which provided spot quality measures, was used to evaluate the quality and reliability of the hybridization. In particular, the flag “glsFound” (set to 1 if the spot had an intensity value significantly different from the local background and to 0 when otherwise) was used to filter out unreliable probes: the flag equal to 0 was to be noted as “not available (NA).” Probes with a high proportion of NA values were removed from the dataset in order to carry out a more solid and unbiased statistical analyses. Thirty-five percent of NA was used as the threshold in the filtering process, and a total of 27,335 krill transcripts were obtained. Principal Component Analysis (PCA), cluster analysis, and profile similarity searches were performed with MultiExperiment Viewer version 4.8.1 (tMev) of the TM4 Microarray Software Suite [63]. Differentially expressed genes were identified using a one-way ANOVA test and the Adjusted p-value was ≤ 0.05 (FDR ≤ 0.05). The normalized expression values of the four biological replicates collected at each time point were log2 transformed and mediated. Values with a coefficient of variation (defined as the ratio of the standard deviation to the mean) > 0.5 were discarded.

CircWaveBatch 3.3 (by courtesy of Dr. Roelof Hut, http://www.euclock.org/) was used to identify genes showing a sinusoidal differential expression during the 24-hour cycle. This statistical algorithm tests each expression profile against a harmonic curve by varying the phase and period length. A F-test was used to measure the goodness-of-fit and the transcript was considered rhythmic when the harmonic regression was statistically significant (p<0.05). Because of the relatively small number of time points assessed here, we decided to set values for the period length parameter as only 12 and 24 hours [64].

### Annotation and functional enrichment of differentially expressed genes

As described in Figure 1B, an automatic bioinformatic tool was developed to annotate isotigs and singletons generated by Newbler 2.5.1. Each consensus, converted into a FASTA format, was searched locally against a nucleotide database, downloaded from NCBI and UniProtUK database, using, respectively, Blast-X and Blast-N. The first 10 High Scoring Pairs (HSPs) from each blast were automatically examined and matches with expectation values greater than e^−6^ for protein (Blast-X) and e^−50^ for nucleotide (Blast-N) were discarded as they were considered poorly informative. The algorithm, moreover, gave priority to the Blast-N hits and discarded alignments characterized by less than 30% of coverage. With regard to the five best hits, the software selected the description related to the organism with the closest taxonomic distance with respect to *E. superba*. The annotation of differentially expressed isotigs and singletons obtained from microarray experiments was also examined manually. GO analysis of differentially expressed genes was performed using the DAVID tool [65].

### Opsin phylogenetic analysis

*E. superba* opsin cDNA sequences were converted into amino acid sequences using the translate tool by ExPASy Proteomics (http://www.expasy.org/tools/dna.html) and then aligned with other crustacean opsin sequences obtained from GenBank using ClustalW2 [66]. A phylogenetic tree was generated using a neighbor-joining algorithm with Dayhoff correction (MEGA 4.0; [67]). The bootstrap test (1,000 replicates) was performed. A pairwise deletion algorithm was also used to eliminate any alignment gaps present in the sequence. The tree was rooted using the *Bos taurus* rhodopsin as outgroup.

### Validation of relative gene expression by quantitative RT-PCR

Quantitative RT-PCR was used to validate the expression values of nine differentially expressed genes obtained from microarray experiments. 1 μg of total RNA from the head of single specimens was used to perform independent cDNA syntheses in a final volume of 10 μl, using random hexamers and SuperScript II reverse transcriptase (Life Technologies). Three biological replicates were analyzed. One μl aliquot of 1∶100 diluted first-strand cDNA was PCR amplified in 10 μl volume using the SYBR Green chemistry according to the manufacturer's recommendations (GoTaq qPCR Master Mix, Promega). Gene-specific primers (Table S2) were designed using the Primer3 desgn tool [68] to obtain 60–80 bp amplicons. To avoid the amplification of contaminant genomic DNA, total RNA samples were treated with DNase I (Qiagen, Gaithersburg, MD, USA). A dissociation curve was used to confirm the specificity of the amplicon. We verified the efficiency of the primers by drawing standard curves for target genes and endogenous control (*18S rRNA*). PCR reactions were performed in triplicate in a 7500 Real-Time PCR System (Applied Biosystems). Thermal cycling conditions were as follows: 2 min denaturation at 95°C followed by 38 cycles for 25 sec denaturation at 95°C, 1 min annealing and elongation at 60°C, and a final 3 min elongation at 72°C. The 2^−ΔΔCt^ (RQ, relative quantification) method implemented in the 7500 Real Time PCR System software was used to calculate the relative expression ratio [69]. This method defines the change in expression of a nucleic acid sequence (target) in test samples (krill collected at 01∶00, 06∶00, 10∶00, 15∶00, and 18∶00 h) relative to the same sequence in a calibrator sample considered the fishing time when the target gene is expressed at its lowest levels. 95% confidence intervals are associated to each time point. Pearson's correlation was calculated to estimate the association between not mediated microarray data and qRT-PCR results.

## Supporting Information

Figure S1 Flow chart of the assembly and automated annotation of 454 reads. **A)** **Assembly of 454 reads.** Raw reads: chromatograms produced by 454 Titanium sequencing; Trimmed reads: reads processed for assembling; Singletons: putative transcripts identified by one read; Singletons > 200 nt: putative transcripts identified by one read > 200 nucleotide length; Isotigs: putative transcripts identified by at least two reads; Consensus sequences: non-redundant sequences (singletons + isotigs). **B) Automated annotation process.** Each consensus sequence, converted to the FASTA format, was searched locally against a nucleotide database downloaded from the NCBI and UniProtUK databases using, respectively, Blast-X and Blast-N. See Material and Methods for more details.
(TIF)

Figure S2 Functional analysis of differentially expressed transcripts. **A)** A weighted Venn diagram showing the relative portion in the differentially expressed genes of those with sinusoidal expression patterns (CircWaveBatch analysis). **B)** The differentially expressed annotated genes (336 consensus sequences) were classified into 12 different functional categories. The diagram shows the proportion of each functional category. **C)** The 159 annotated transcripts showing sinusoidal oscillatory patterns were grouped into 12 functional categories. Transcripts characterized by a 24-hour (75 out of 159) or a 12-hour (84 out of 159) periodicity of expression are shown separately. See Table S1 for more details.
(TIF)

Figure S3 Validation of microarray expression values by qRT-PCR. mRNA expression levels are represented by box-and-whisker plots. Normalized qRT-PCR data are expressed as fold changes (FC) relative to the median expression for each time point. *18S rRNA* was used as an endogenous control. The microarray expression profile of each gene is shown below the qRT-PCR box plot. Pearson's correlation was calculated to estimate the association between the microarray data and qRT-PCR results (p > 0.7 is considered statistically significant).
(TIF)

Figure S4 Schematic representation of a normalized cDNA library construction protocol. A combination of two different protocols – the “whole transcriptome amplification (WTA)” and “Duplex-specific nuclease (DSN) normalization” – was adopted. See Material and Methods for more details.
(TIF)

Table S1 The list of 336 differentially expressed annotated genes grouped into functional categories. Expression levels over a 24-hour cycle are shown. Transcripts identified as cycling by CircWaveBatch V. 3.3 are indicated in bold. Sampling times are indicated. ^a^Annotation  =  description of the gene; ^b^e-value: score of annotation with Blast-N and/or Blast-X; ^c^Probe: ID of probe sequence in “Krill 1.1” Agilent microarray platform.
(PDF)

Table S2 Primers used in quantitative RT-PCR.
(PDF)
