# Supplementary figures and images for: The Antarctic Krill Euphausia superba Shows Diurnal Cycles of Transcription under Natural Conditions
Source: PLoS One. 2013 Jul 17;8(7):e68652. doi: 10.1371/journal.pone.0068652 (PMC3714250; doi:10.1371/journal.pone.0068652)

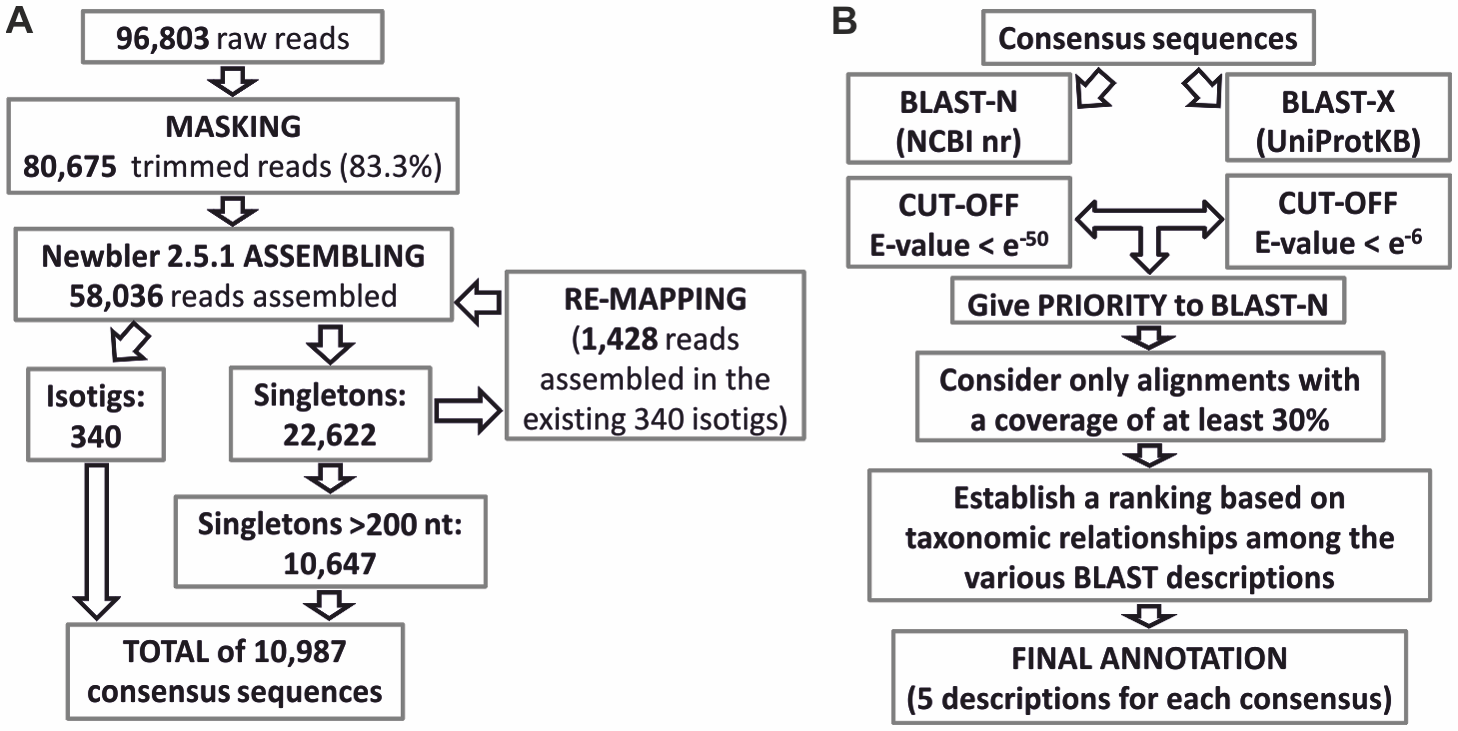

Supplement: Figure S1 — Flow chart of the assembly and automated annotation of 454 reads. A) Assembly of 454 reads. Raw reads: chromatograms produced by 454 Titanium sequencing; Trimmed reads: reads processed for assembling; Singletons: putative transcripts identified by one read; Singletons > 200 nt: putative transcripts identified by one read > 200 nucleotide length; Isotigs: putative transcripts identified by at least two reads; Consensus sequences: non-redundant sequences (singletons + isotigs). B) Automated annotation process. Each consensus sequence, converted to the FASTA format, was searched locally against a nucleotide database downloaded from the NCBI and UniProtUK databases using, respectively, Blast-X and Blast-N. See Material and Methods for more details. (TIF) [file pone.0068652.s001.tif]

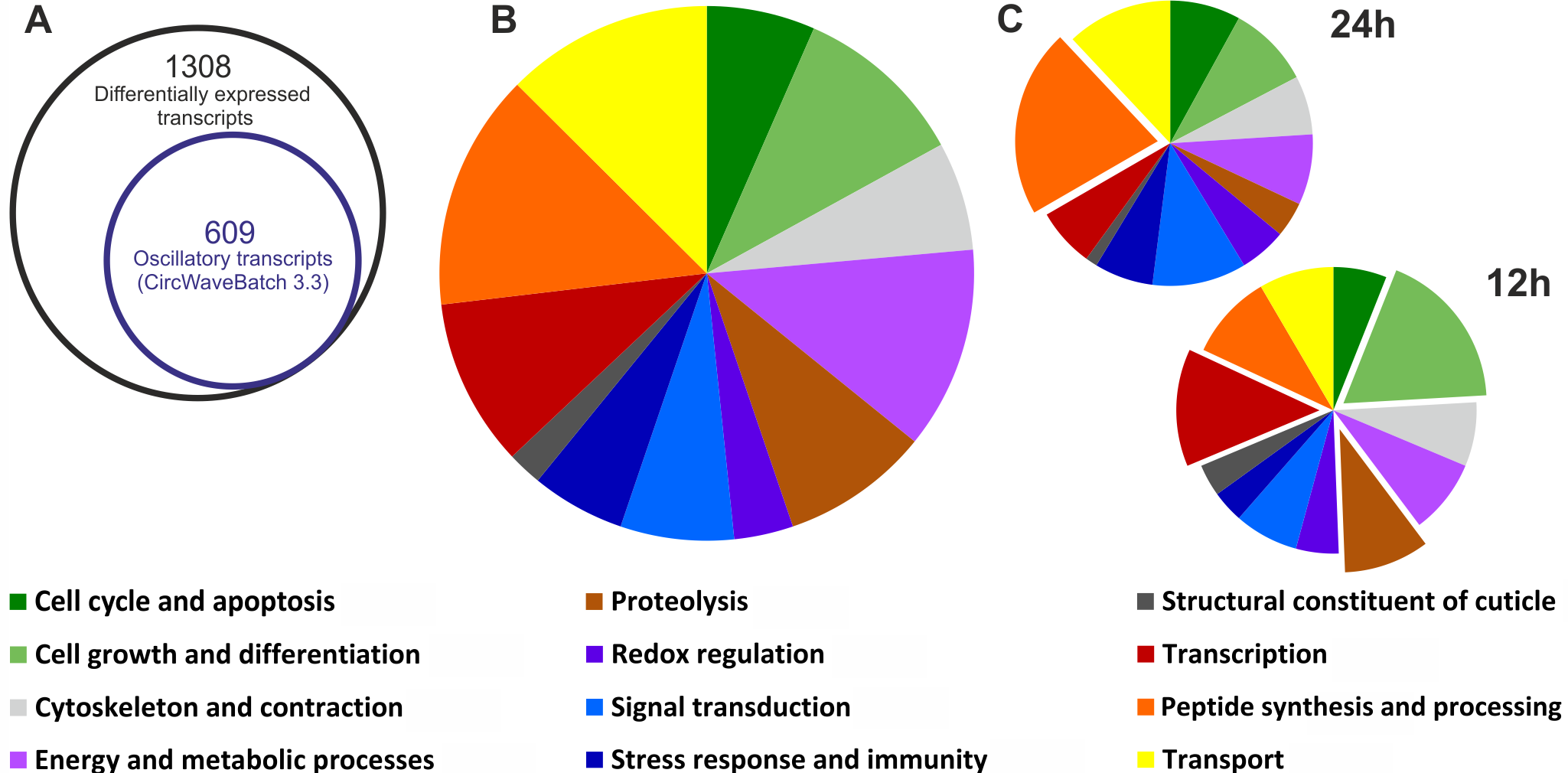

Supplement: Figure S2 — Functional analysis of differentially expressed transcripts. A) A weighted Venn diagram showing the relative portion in the differentially expressed genes of those with sinusoidal expression patterns (CircWaveBatch analysis). B) The differentially expressed annotated genes (336 consensus sequences) were classified into 12 different functional categories. The diagram shows the proportion of each functional category. C) The 159 annotated transcripts showing sinusoidal oscillatory patterns were grouped into 12 functional categories. Transcripts characterized by a 24-hour (75 out of 159) or a 12-hour (84 out of 159) periodicity of expression are shown separately. See Table S1 for more details. (TIF) [file pone.0068652.s002.tif]

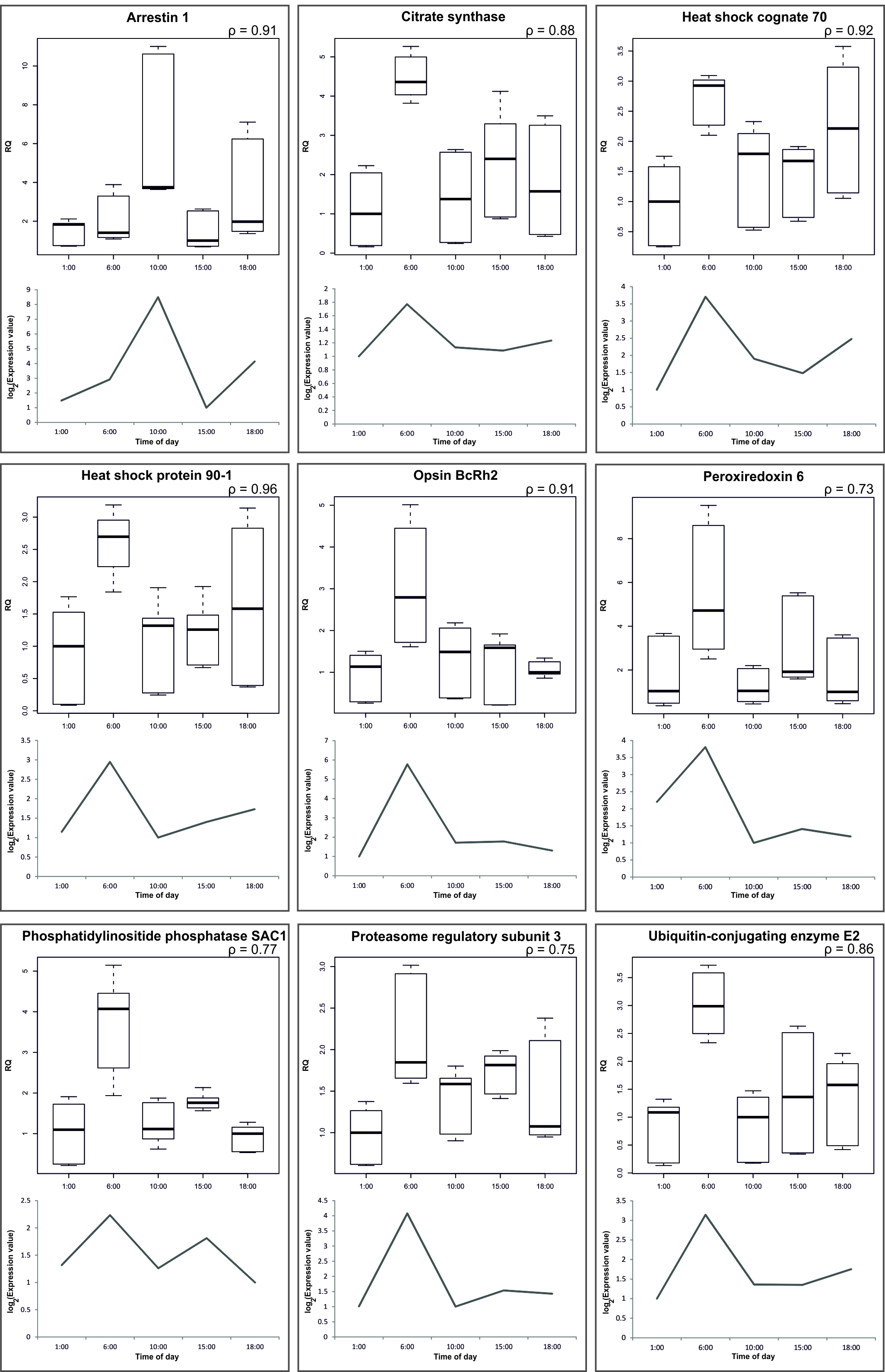

Supplement: Figure S3 — Validation of microarray expression values by qRT-PCR. mRNA expression levels are represented by box-and-whisker plots. Normalized qRT-PCR data are expressed as fold changes (FC) relative to the median expression for each time point. 18S rRNA was used as an endogenous control. The microarray expression profile of each gene is shown below the qRT-PCR box plot. Pearson's correlation was calculated to estimate the association between the microarray data and qRT-PCR results (p > 0.7 is considered statistically significant). (TIF) [file pone.0068652.s003.tif]

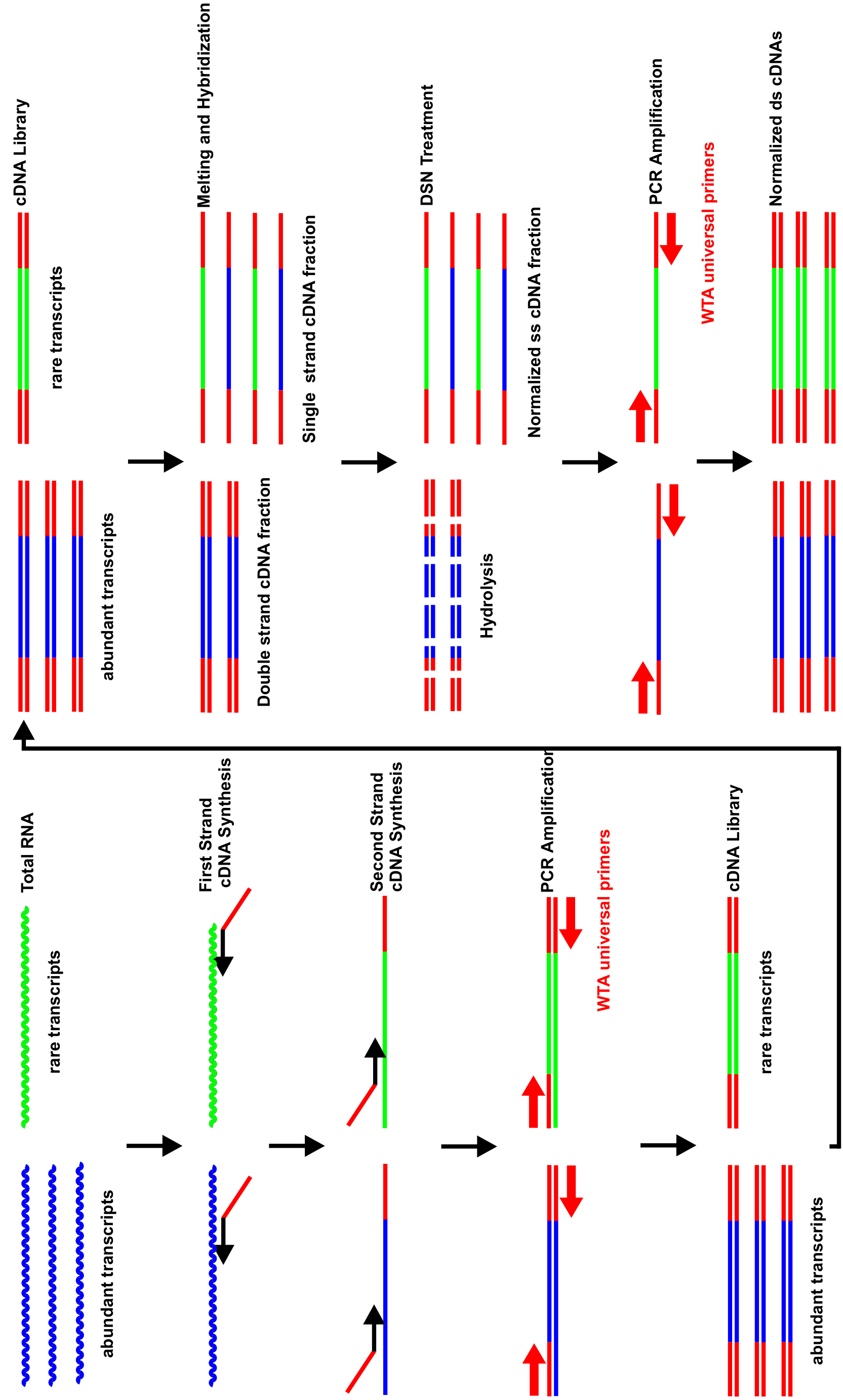

Supplement: Figure S4 — Schematic representation of a normalized cDNA library construction protocol. A combination of two different protocols – the “whole transcriptome amplification (WTA)” and “Duplex-specific nuclease (DSN) normalization” – was adopted. See Material and Methods for more details. (TIF) [file pone.0068652.s004.tif]
