# Supplementary material for: The Antarctic Krill Euphausia superba Shows Diurnal Cycles of Transcription under Natural Conditions
Source: PLoS One. 2013 Jul 17;8(7):e68652. doi: 10.1371/journal.pone.0068652 (PMC3714250; doi:10.1371/journal.pone.0068652)
Supplement: Table S1 — The list of 336 differentially expressed annotated genes grouped into functional categories. Expression levels over a 24-hour cycle are shown. Transcripts identified as cycling by CircWaveBatch V. 3.3 are indicated in bold. Sampling times are indicated. aAnnotation = description of the gene; be-value: score of annotation with Blast-N and/or Blast-X; cProbe: ID of probe sequence in “Krill 1.1” Agilent microarray platform. (PDF) [file pone.0068652.s005.pdf]

Functional category	τ	Annotation[a]	e-value[b]	1:00	6:00	10:00	15:00	18:00	Probe[c]
**Cell cycle and Apoptosis**	24	**Bax inhibitor-1-like protein (*T. castaneum*)**	2.00E-53	8.28	9.23	7.59	8.14	7.92	CUST_5802_PI426442911
	24	**programmed cell death protein 4 (*H. sapiens*)**	8.06E-11	6.05	6.75	5.40	5.83	5.31	CUST_23348_PI426442911
	24	**tyrosine kinase (*I. scapularis*)**	4.00E-42	4.72	5.25	4.62	4.04	4.30	CUST_27279_PI426442911
	24	**protein JTB (*B. malayi*)**	1.65E-13	6.47	7.36	6.65	6.73	6.18	CUST_10717_PI426442911
	24	**Mitogen-activated protein kinase pmk-1 (*C. elegans*)**	5.00E-47	6.65	6.96	7.26	6.92	6.28	CUST_23182_PI426442911
	24	**apoptosis regulator Bcl-X (*H. saltator*)**	1.05E-21	10.60	10.72	11.07	10.90	11.06	CUST_6184_PI426442911
	12	**serine/threonine-protein kinase kinX (*D. discoideum*)**	4.42E-13	8.67	7.94	8.45	8.77	8.12	CUST_17478_PI426442911
	12	**kynureninase (*M. brevicollis*)**	1.02E-15	4.23	4.69	3.17	5.14	2.76	CUST_21653_PI426442911
	12	**apoptosis-stimulating of p53 protein 1 (*M. musculus*)**	2.62E-27	7.53	7.58	7.90	7.24	7.75	CUST_13537_PI426442911
	12	**deoxyribonuclease Tat-D (*S. pombe*)**	6.70E-15	4.38	4.01	5.00	4.26	4.17	CUST_32189_PI426442911
	12	**protein kinase C-like 1B (*C. elegans*)**	2.16E-13	3.92	4.52	6.19	4.37	4.54	CUST_26566_PI426442911
		MOB kinase activator 3A (*M. musculus*)	5.00E-15	5.55	4.53	3.52	4.39	4.17	CUST_24781_PI426442911
		S-adenosylmethionine synthase 5 (*C. elegans*)	1.40E-137	7.26	7.21	6.28	6.99	6.44	CUST_1489_PI426442911
		N-acetyltransferase san-like (*A. mellifera*)	2.00E-76	7.18	7.74	6.39	6.80	6.85	CUST_6102_PI426442911
		pelota (*D. melanogaster*)	3.00E-110	7.95	8.77	7.82	7.78	8.02	CUST_6915_PI426442911
		S-adenosylmethionine synthase 4 (*C. elegans*)	1.55E-98	7.64	7.86	6.67	7.59	7.02	CUST_1488_PI426442911
		serine/threonine-protein kinase 24 (*A. pisum*)	5.00E-08	7.78	8.39	7.19	7.38	7.24	CUST_6673_PI426442911
		transmembrane BAX inhibitor motif-containing protein 1 (*S. salar*)	7.00E-42	6.04	7.29	4.43	4.54	3.84	CUST_18103_PI426442911
		zinc finger CCCH domain-containing protein 15 homolog (*C. briggsae*)	1.61E-89	7.26	7.63	6.98	7.45	6.73	CUST_6553_PI426442911
		putative DNA helicase INO80 complex 1 (*M. musculus*)	1.31E-18	6.36	5.95	6.38	6.35	6.33	CUST_10783_PI426442911
		short spindle protein 4 (*D. melanogaster*)	2.14E-63	9.27	9.12	9.73	9.47	9.52	CUST_5762_PI426442911
		structural maintenance of chromosomes protein 2 (*X. laevis*)	6.97E-20	3.07	3.81	3.23	4.05	4.59	CUST_28148_PI426442911
**Cell growth and differentiation**	24	**reticulon 4 interacting protein 1 (*D. rerio*)**	2.00E-23	5.62	6.29	5.68	5.62	5.52	CUST_5330_PI426442911
	24	**cytoplasmic dynein 1 light intermediate chain 2 (*C. floridanus*)**	1.00E-56	4.14	5.50	4.76	4.64	4.50	CUST_28615_PI426442911
	24	**1-acyl-SN-glycerol-3-phosphate acyltransferase (*R. loti*)**	2.25E-33	2.22	2.88	4.12	3.18	3.16	CUST_31360_PI426442911
	24	**Ras GTPase-activating protein 3 (*B. taurus*)**	1.32E-18	6.22	5.75	6.60	6.64	6.32	CUST_28690_PI426442911
	24	**UDP-N-acetylglucosamine--peptide N-acetylglucosaminyltransferase (*C. elegans*)**	8.25E-48	6.15	6.06	6.73	6.48	6.59	CUST_13268_PI426442911
	24	**vitellogenin (*F. merguiensis*)**	3.50E-20	13.80	13.40	14.58	14.51	14.89	CUST_15830_PI426442911
	12	**la-related protein (*D. melanogaster*)**	3.34E-27	6.68	5.77	6.17	6.40	6.10	CUST_7510_PI426442911
	12	**zinc finger RAD18 domain-containing protein C1orf124 (*H. sapiens*)**	1.87E-13	5.88	5.18	5.44	5.59	4.92	CUST_18415_PI426442911
	12	**trimethyllysine dioxygenase, mitochondrial (*C. quinquefasciatus*)**	0.00E+00	2.27	2.22	2.03	2.62	2.01	CUST_22849_PI426442911
…									

Functional category	τ	Annotation[a]	e-value[b]	1:00	6:00	10:00	15:00	18:00	Probe[c]
… **Cell growth and differentiation**	12	**ankyrin 2 (*C. quinquefasciatus*)**	1.24E-16	7.96	8.33	8.09	7.87	8.50	CUST_18742_PI426442911
	12	**cortactin-binding protein 2 (*H. glaber*)**	2.00E-05	4.90	5.08	5.70	4.56	5.06	CUST_14110_PI426442911
	12	**juvenile hormone esterase binding protein (*M. sexta*)**	5.00E-09	5.49	5.16	6.46	5.19	6.03	CUST_15902_PI426442911
	12	**basement membrane-specific heparan sulfate proteoglycan core protein (*A. echinatior*)**	0.00E+00	3.19	2.64	3.97	2.32	3.31	CUST_12056_PI426442911
	12	**LIM class homeobox transcription factor Lmx (*M. leidyi*)**	4.35E-30	8.67	8.16	9.42	8.62	8.72	CUST_7592_PI426442911
	12	**nuclear hormone receptor family member nhr-48 (*C. elegans*)**	2.73E-28	4.33	4.17	5.06	4.27	4.35	CUST_15463_PI426442911
	12	**ORM1-like protein 1 (*X. laevis*)**	9.20E-26	3.95	3.97	5.30	3.21	2.53	CUST_10938_PI426442911
	12	**phosphatidylinositol synthase (*A. aegypti*)**	1.0E-45	5.03	4.56	5.84	4.90	5.48	CUST_23947_PI426442911
	12	**ribose-phosphate pyrophosphokinase 3 (*S. pombe*)**	9.49E-38	4.28	3.69	4.52	3.22	4.11	CUST_10236_PI426442911
	12	**structural maintenance of chromosomes protein 6 (*D. discoideum*)**	3.19E-46	6.30	6.32	6.65	6.22	6.11	CUST_7326_PI426442911
	12	**univin precursor (*P. humanus corporis*)**	6.00E-08	4.25	3.60	4.90	4.20	4.01	CUST_22578_PI426442911
	12	**ureidoglycolate lyase (*B. pseudomallei*)**	9.01E-20	5.03	4.90	6.41	5.35	5.46	CUST_22089_PI426442911
		1-acyl-sn-glycerol-3-phosphate acyltransferase 2 (*B. oleracea*)	8.06E-62	8.92	9.54	8.50	8.42	8.91	CUST_5736_PI426442911
		cleft lip and palate transmembrane protein 1 homolog (*B. taurus*)	3.72E-43	5.65	6.09	5.23	5.63	5.03	CUST_16781_PI426442911
		dynein heavy chain, cytoplasmic-like (*A. florea*)	1.00E-105	7.49	7.77	6.71	7.11	7.30	CUST_6948_PI426442911
		farnesoic acid O-methyltransferase (*P. monodon*)	2.38E-105	7.80	7.98	6.79	7.86	7.10	CUST_6310_PI426442911
		nicalin-1 (*A. mellifera*)	6.00E-30	4.01	5.40	3.87	3.90	4.04	CUST_7137_PI426442911
		transmembrane emp24 domain-containing protein 3 (*L. salmonis*)	1.78E-18	4.94	5.68	4.91	5.19	4.14	CUST_22952_PI426442911
		Hyccin (*H. saltator*)	5.47E-11	9.06	8.71	9.34	9.11	8.97	CUST_11140_PI426442911
		nipped-B-like protein pqn-85 (*C. elegans*)	1.32E-38	8.22	7.86	8.62	8.51	8.35	CUST_6151_PI426442911
		protein kinase C-binding protein NELL1-like isoform 1 (*B. terrestris*)	2.00E-17	5.24	5.02	6.05	5.31	5.72	CUST_11850_PI426442911
		putative neural-cadherin 2 (*D. melanogaster*)	4.10E-33	6.25	6.03	6.74	6.32	6.47	CUST_23121_PI426442911
		sex determination protein fruitless (*D. melanogaster*)	6.30E-13	1.95	2.11	3.62	2.53	2.25	CUST_21055_PI426442911
		protein 4.1 homolog (*D. melanogaster*)	2.14E-29	8.18	8.09	8.20	8.28	7.66	CUST_23602_PI426442911
**Cytoskeleton and contraction**	24	**dynein light chain roadblock-type 2-like (*B. terrestris*)**	2.00E-46	4.30	4.91	2.62	3.65	3.59	CUST_6718_PI426442911
	24	**myosin-J heavy chain (*D. discoideum*)**	5.02E-51	3.58	3.33	3.06	2.66	2.08	CUST_4216_PI426442911
	24	**similar to reticulocalbin (*T. castaneum*)**	2.00E-34	5.06	5.72	4.94	4.79	4.63	CUST_16912_PI426442911
	24	**myosin iii (*C. quinquefasciatus*)**	9.14E-11	10.01	10.35	10.33	9.67	9.80	CUST_13341_PI426442911
	24	**alpha actin (*D. dorsalis*)**	0	7.96	5.34	5.66	5.98	5.72	CUST_3204_PI426442911
	12	**FERM domain-containing protein 4A (*M. musculus*)**	2.81E-21	6.69	5.92	6.25	6.26	6.12	CUST_11356_PI426442911
	12	**troponin I (*P. leptodactylus*)**	2.57E-44	7.89	6.38	6.76	7.10	6.67	CUST_4405_PI426442911
…									

Functional category	τ	Annotation[a]	e-value[b]	1:00	6:00	10:00	15:00	18:00	Probe[c]
…<br>**Cytoskeleton and contraction**	12	**77 kDa echinoderm microtubule-associated protein (*S. purpuratus*)**	1.08E-31	6.48	7.45	6.19	7.00	6.89	CUST_31723_PI426442911
	12	**filamin-B (*O. cuniculus*)**	3.51E-32	6.31	7.59	6.12	7.01	6.87	CUST_27311_PI426442911
	12	**muscle-specific protein 300, isoform D (*D. melanogaster*)**	5.00E-08	6.12	6.36	6.64	5.75	6.72	CUST_30098_PI426442911
	12	**phosphatidylinositol-4-phosphate 5-kinase type-1 alpha (*C. floridanus*)**	9.77E-28	6.71	6.04	6.75	6.52	6.55	CUST_26929_PI426442911
		myosin-1 (*C. elegans*)	1.50E-11	9.28	9.15	8.52	9.28	7.89	CUST_3352_PI426442911
		actin (*H. thorelli*)	4.00E-56	10.81	11.52	9.81	10.79	10.11	CUST_303_PI426442911
		actin 2 (*P. monodon*)	4.00E-150	10.02	10.89	9.59	9.86	9.85	CUST_9920_PI426442911
		actin, gamma 1 (*H. sapiens*)	0	10.66	11.84	10.10	10.46	10.47	CUST_6106_PI426442911
		actin, gamma 2, smooth muscle (*H. sapiens*)	0	10.00	10.63	8.86	9.61	9.37	CUST_256_PI426442911
		Actr1b protein (*M. musculus*)	2.00E-58	5.32	6.40	4.53	5.18	5.38	CUST_5796_PI426442911
		cytoplasmic actin (*I. confusus*)	1.00E-55	6.70	7.13	5.80	6.57	5.86	CUST_9940_PI426442911
		microtubule-associated proteins 1A/1B light chain 3A (*S. salar*)	3.00E-38	5.48	6.14	4.83	5.17	5.11	CUST_4312_PI426442911
		profilin (*P. monodon*)	8.00E-18	9.16	10.14	8.76	9.14	9.23	CUST_5549_PI426442911
		filamin-A (*H. sapiens*)	2.91E-18	5.29	5.90	4.94	6.88	4.42	CUST_20884_PI426442911
		myosin light chain kinase (*H. sapiens*)	2.00E-10	5.53	5.18	5.90	6.08	5.27	CUST_12091_PI426442911
**Energy and metabolic process**	24	**acetyl-CoA acetyltransferase (*P. denitrificans*)**	2.63E-85	6.74	7.37	5.20	6.24	5.76	CUST_4177_PI426442911
	24	**electron transfer flavoprotein subunit beta (*S. paramamosain*)**	4.00E-98	6.30	6.69	5.84	5.87	5.75	CUST_5909_PI426442911
	24	**short-chain specific acyl-CoA dehydrogenase (*B. impatiens*)**	3.00E-160	4.63	4.59	4.50	3.63	3.23	CUST_5403_PI426442911
	24	**isocitrate dehydrogenase [NAD] subunit beta, mitochondrial (*A. echinatior*)**	2.00E-145	4.33	5.33	4.03	4.01	4.05	CUST_5876_PI426442911
	24	**xylose isomerase (*D. pulex*)**	2.00E-24	3.17	5.43	4.65	4.07	4.25	CUST_7074_PI426442911
	24	**cytochrome c oxidase subunit I (*E. pacifica*)**	0	13.48	13.88	14.36	14.57	13.89	CUST_697_PI426442911
	12	**glycogen synthase (*C. elegans*)**	4.54E-40	4.82	3.73	4.03	4.27	3.58	CUST_20381_PI426442911
	12	**alpha-N-acetylgalactosaminidase (*M. musculus*)**	2.00E-39	4.99	4.33	4.72	5.35	4.09	CUST_28997_PI426442911
	12	**ATP synthase b (*S. exigua*)**	4.00E-87	8.01	9.24	7.50	8.45	8.40	CUST_5502_PI426442911
	12	**glycogen debranching enzyme-like (*N. vitripennis*)**	8.00E-17	8.07	8.60	8.77	7.22	9.12	CUST_11785_PI426442911
	12	**ATP citrate lyase subunit beta-like protein (*S. purpuratus*)**	0.00E+00	2.03	2.35	2.70	2.01	2.20	CUST_18451_PI426442911
	12	**cytochrome b-c1 complex subunit 7 (*B. taurus*)**	3.43E-11	4.46	4.37	5.69	4.30	5.21	CUST_4132_PI426442911
	12	**acetyl-CoA carboxylase (*G. gallus*)**	4.36E-11	4.48	4.41	5.21	4.27	4.43	CUST_30052_PI426442911
		F1F0-ATP synthase beta subunit mRNA (*M. japonicus*)	0	11.48	12.44	11.45	11.63	11.74	CUST_5385_PI426442911
		3-hydroxyacyl-CoA dehydrogenase (*C. elegans*)	3.03E-97	3.62	5.25	3.41	3.94	3.56	CUST_6393_PI426442911
		3-ketoacyl-CoA thiolase (*P. profundum*)	1.65E-64	5.91	6.73	4.09	5.85	5.19	CUST_5383_PI426442911
…									

Functional category	τ	Annotation[a]	e-value[b]	1:00	6:00	10:00	15:00	18:00	Probe[c]
… **Energy and metabolic process**		Acyl-CoA-binding domain-containing protein 6 (*C. floridanus*)	7.00E-57	5.67	6.70	5.20	5.89	5.79	CUST_5434_PI426442911
		ATP synthase subunit d (*A. aegypti*)	1.00E-104	7.01	7.85	6.31	6.71	6.68	CUST_5975_PI426442911
		citrate synthase (*A. aegypti*)	0.00E+00	10.04	10.84	9.99	9.99	10.20	CUST_3897_PI426442911
		cytochrome c oxidase subunit 6a polypeptide 1 (*X. laevis*)	1.00E-30	8.15	9.00	7.47	7.89	7.64	CUST_3868_PI426442911
		cytochrome c oxidase subunit Va (*A. yamamai*)	0	7.93	8.65	6.84	7.89	7.50	CUST_7744_PI426442911
		cytosolic malate dehydrogenase (*D. pulex*)	1.00E-141	9.66	10.73	8.46	9.34	9.15	CUST_5902_PI426442911
		electron transfer flavoprotein subunit alpha (*S. salar*)	2.00E-110	6.56	7.63	5.35	6.35	5.84	CUST_6213_PI426442911
		F1F0-ATP synthase beta subunit (*M. japonicus*)	97	11.59	12.56	11.48	11.72	11.80	CUST_5384_PI426442911
		fructose 1,6-bisphosphatase (*M. japonicus*)	0	6.77	6.82	5.68	6.74	5.16	CUST_6390_PI426442911
		glyceraldehyde-3-phosphate dehydrogenase (*G. gracilis*)	0	11.94	13.14	12.05	11.89	11.94	CUST_5467_PI426442911
		Glycogenin-1 (*S. cerevisiae*)	1.98E-26	3.34	4.12	2.20	2.98	2.05	CUST_5589_PI426442911
		malate dehydrogenase, mitochondrial (*D. rerio*)	2.00E-96	7.58	9.33	6.85	7.71	7.29	CUST_1883_PI426442911
		malateÿdehydrogenaseÿ(*S. paramamosain*)	0	7.48	9.24	6.91	7.78	7.33	CUST_1878_PI426442911
		malic enzyme (*C. quinquefasciatus*)	3.00E-158	6.59	7.82	5.55	6.14	6.77	CUST_5731_PI426442911
		NADH dehydrogenase (ubiquinone) Fe-S protein 4 (*T. castaneum*)	5.00E-50	7.53	8.53	6.82	7.38	7.38	CUST_8124_PI426442911
		NADH:ubiquinone oxidoreductase ESSS subunit (*D. plexippus*)	1.00E-15	6.87	7.67	6.03	6.57	6.81	CUST_3138_PI426442911
		NADH-ubiquinone oxidoreductase fe-s protein 2 (*A. aegypti*)	0	6.11	7.14	5.34	5.65	5.97	CUST_5913_PI426442911
		phosphopantothenoylcysteine decarboxylase subunit SIS2 (*S. cerevisiae*)	2.01E-11	3.93	5.11	3.40	4.71	3.96	CUST_18531_PI426442911
		phosphopyruvate hydratase (*P. monodon*)	0	8.72	9.87	8.17	8.62	8.81	CUST_1501_PI426442911
		pyruvate kinase-like isoform 1 (*A. pisum*)	1.00E-07	7.39	8.55	6.26	6.99	7.04	CUST_16780_PI426442911
		succinate dehydrogenase (*C. quinquefasciatus*)	2.00E-25	5.15	6.20	4.72	5.14	4.87	CUST_5997_PI426442911
		transketolase-like protein 1 (*B. taurus*)	3.16E-33	8.77	9.07	7.19	8.10	8.14	CUST_3906_PI426442911
		translational activator of cytochrome c oxidase 1-like (*N. vitripennis*)	1.00E-08	4.23	4.53	3.37	4.39	3.22	CUST_32119_PI426442911
		6-phosphofructokinase (*S. mansoni*)	7.51E-39	5.19	5.64	5.48	5.65	4.74	CUST_15522_PI426442911
		glycogen debranching enzyme-like (*B. terrestris*)	2.00E-09	9.56	9.87	9.35	8.57	10.43	CUST_12153_PI426442911
**Peptide synthesis and processing**	24	**aspartate beta-hydroxylase domain-containing protein 2 (*X. tropicalis*)**	9.48E-23	4.30	3.45	2.92	3.36	2.00	CUST_6616_PI426442911
	24	**pentatricopeptide repeat-containing protein (*I. scapularis*)**	0.00E+00	4.82	3.63	3.70	3.30	3.40	CUST_28270_PI426442911
	24	**peptidyl-prolyl cis-trans isomerase FKBP14 (*P. abelii*)**	3.26E-31	6.39	7.42	5.70	6.30	5.59	CUST_5455_PI426442911
	24	**adaptor protein, phosphotyrosine interaction, PH domain and leucine zipper containing 1 (*T. guttata*)**	9.00E-10	6.39	7.14	6.32	6.07	5.66	CUST_21238_PI426442911
	24	**COP9 signalosome complex subunit 1 (*D. rerio*)**	3.00E-43	4.63	5.63	4.31	4.41	4.17	CUST_15998_PI426442911
	24	**tRNA (guanine-N(7)-)-methyltransferase (*B. malayi*)**	1.67E-51	4.77	5.03	4.34	4.75	3.81	CUST_9981_PI426442911
…									

Functional category	τ	Annotation[a]	e-value[b]	1:00	6:00	10:00	15:00	18:00	Probe[c]
… **Peptide synthesis and processing**	24	**glutamyl-prolyl-tRNA synthetase (*D. rerio*)**	4.00E-17	3.45	4.50	3.90	3.34	3.44	CUST_21928_PI426442911
	24	**MAP kinase-interacting serine/threonine-protein kinase 1-like (*A. florea*)**	2.00E-75	4.82	5.98	5.30	4.87	5.07	CUST_24265_PI426442911
	24	**tRNA 2-thiocytidine biosynthesis protein TtcA (*S. pomeroyi*)**	2.77E-13	2.34	3.44	2.00	2.36	2.04	CUST_23468_PI426442911
	24	**ubiquitin related modifier 1 (*X. tropicalis*)**	7.00E-22	3.66	4.62	3.70	3.68	3.38	CUST_27605_PI426442911
	24	**translation initiation factor IF-3 (*A. aurescens*)**	0	9.07	10.16	9.67	9.52	9.18	CUST_7275_PI426442911
	24	**myotubularin-related protein 4 (*X. laevis*)**	3.12E-12	4.71	5.30	5.68	5.36	5.17	CUST_22158_PI426442911
	24	**CDKAL1-like protein (*C. elegans*)**	4.93E-40	3.75	3.42	4.48	5.51	4.11	CUST_26804_PI426442911
	24	**peptidyl-prolyl cis-trans isomerase 1 (*B. malayi*)**	7.36E-14	4.29	3.56	4.16	4.35	5.36	CUST_10751_PI426442911
	12	**ribosomal protein L3 (*L. testaceipes*)**	1.05E-51	4.77	4.49	3.36	5.07	3.85	CUST_3778_PI426442911
	12	**luminal-binding protein 1 (*D. discoideum*)**	3.35E-59	5.69	6.11	5.06	6.16	5.43	CUST_3169_PI426442911
	12	**translation initiation factor eIF-2B subunit delta (*D. discoideum*)**	6.73E-13	5.11	5.68	4.73	5.34	5.37	CUST_26251_PI426442911
	12	**60S ribosomal protein L34 (*D. discoideum*)**	7.47E-29	12.15	12.97	12.49	12.15	12.60	CUST_3151_PI426442911
	12	**tubulin-specific chaperone D (*M. musculus*)**	1.35E-23	7.63	9.65	7.71	7.07	8.86	CUST_27042_PI426442911
	12	**type II hemocyte transglutaminase (*L. vannamei*)**	2.66E-11	8.74	9.75	9.21	7.91	9.85	CUST_12646_PI426442911
	12	**glucosyl/glucuronosyl transferases (*A. aegypti*)**	9.00E-23	4.25	4.26	4.72	3.64	4.41	CUST_16257_PI426442911
	12	**eukaryotic translation initiation factor 3 subunit G (*N. vectensis*)**	1.42E-45	3.26	3.32	4.28	2.87	2.28	CUST_8839_PI426442911
	12	**protein pxr1 (*S. pombe*)**	2.73E-15	6.86	6.90	7.36	6.93	6.85	CUST_6917_PI426442911
		ribosomal RNA methyltransferase nop2 (*S. pombe*)	2.55E-45	4.48	3.61	4.02	2.92	2.79	CUST_8638_PI426442911
		40S ribosomal protein S4 (*I. scapularis*)	2.03E-116	6.41	7.04	5.99	6.40	6.21	CUST_3797_PI426442911
		carbohydrate sulfotransferase 11 (*C. floridanus*)	2.17E-12	5.29	5.55	4.56	5.11	4.91	CUST_27867_PI426442911
		dolichyl-diphosphooligosaccharide--protein glycosyltransferase subunit DAD1 (*A. ventricosus*)	1.28E-41	7.60	8.44	6.54	7.92	6.59	CUST_5623_PI426442911
		elongation factor-1 alpha (*C. gracilis*)	0	11.22	12.55	11.37	11.45	11.65	CUST_6107_PI426442911
		glucose-regulated protein 78 (*F. chinensis*)	1.03E-108	9.33	10.01	8.58	9.25	8.82	CUST_5782_PI426442911
		mitochondrial 28S ribosomal protein S5 (*L. salmonis*)	1.32E-17	4.34	5.45	3.72	4.73	4.42	CUST_8795_PI426442911
		peptidyl-prolyl cis-trans isomerase B (*S. japonicum*)	7.23E-65	8.95	10.01	7.67	9.45	8.18	CUST_3803_PI426442911
		Prefoldin subunit 4 (*O. mykiss*)	3.00E-13	6.40	7.25	5.84	6.17	6.09	CUST_9952_PI426442911
		probable signal peptidase complex subunit 2 (*C. elegans*)	1.04E-24	5.33	6.41	4.34	5.73	4.40	CUST_5462_PI426442911
		protein disulfide-isomerase 2 (*C. elegans*)	7.27E-59	7.98	9.15	7.01	8.48	7.38	CUST_2446_PI426442911
		protein disulfide-isomerase 2 (*C. elegans*)	7.37E-59	7.98	9.16	7.12	8.44	7.50	CUST_2447_PI426442911
		ribos-1 mRNA for ribosome like protein (*M. japonicus*)	0	13.31	13.87	13.45	13.28	13.39	CUST_2937_PI426442911
		ribosomal protein P1 (*P. varians*)	1.00E-27	5.24	6.05	4.72	4.90	5.14	CUST_4985_PI426442911
		ribosome maturation protein SBDS (*S. salar*)	4.00E-86	5.46	6.34	5.28	5.37	5.54	CUST_5450_PI426442911
…									

Functional category	τ	Annotation[a]	e-value[b]	1:00	6:00	10:00	15:00	18:00	Probe[c]
… **Peptide synthesis and processing**		S-adenosylmethionine synthase 5 (*C. elegans*)	2.18E-150	7.55	7.74	6.61	7.41	6.93	CUST_1485_PI426442911
		selenoprotein M (*X. laevis*)	2.52E-21	8.47	9.13	7.54	8.75	7.70	CUST_8115_PI426442911
		signal peptidase complex catalytic subunit sec11 (*D. discoideum*)	6.47E-12	6.32	6.45	5.71	6.22	5.61	CUST_9804_PI426442911
		similar to eukaryotic translation initiation factor (*T. castaneum*)	3.00E-73	6.96	7.92	6.63	6.93	7.12	CUST_2880_PI426442911
		T-complex Chaperonin 5 (*S. paramamosain*)	0	10.45	11.19	9.80	10.07	10.49	CUST_3225_PI426442911
		threonyl-tRNA synthetase (*C. quinquefasciatus*)	1.00E-111	6.27	6.80	5.37	5.98	5.88	CUST_6959_PI426442911
		translation initiation factor eIF3, p35 subunit (*I. scapularis*)	2.00E-11	6.56	7.08	5.39	6.00	5.88	CUST_6924_PI426442911
		tRNA (cytosine-5-)-methyltransferase CG6133 (*D. melanogaster*)	1.04E-13	5.97	6.37	5.56	6.28	5.62	CUST_4822_PI426442911
		dolichyl-diphosphooligosaccharide--protein glycosyltransferase subunit STT3 (*D. discoideum*)	7.77E-49	4.58	4.94	4.55	5.83	4.36	CUST_10037_PI426442911
**Proteolysis**	24	**lysosome-associated membrane glycoprotein 1 (*B. taurus*)**	8.69E-17	9.26	9.34	8.69	8.26	8.91	CUST_5903_PI426442911
	24	**cathepsin L (*P. monodon*)**	6.00E-178	10.56	10.83	10.17	9.81	10.17	CUST_4125_PI426442911
	24	**presenilins-associated rhomboid-like protein (*B. taurus*)**	2.22E-74	5.97	6.39	5.31	5.54	5.30	CUST_6343_PI426442911
	12	**cysteine and histidine-rich protein 1 homolog (*D. melanogaster*)**	2.77E-21	4.85	5.77	4.86	6.09	5.76	CUST_17671_PI426442911
	12	**proteasome 25 kDa subunit (*T. castaneum*)**	2.00E-127	5.58	7.62	5.27	6.31	6.37	CUST_2262_PI426442911
	12	**ubiquitin protein 7 (*B. taurus*)**	4.52E-13	4.50	5.60	3.93	4.35	5.04	CUST_6824_PI426442911
	12	**protein DDI1 homolog 2 (*X. tropicalis*)**	9.27E-17	4.18	4.65	4.93	3.90	4.95	CUST_22750_PI426442911
	12	**ADAM 17-like protease (*A. echinatior*)**	6.00E-25	3.89	3.10	4.52	3.09	3.71	CUST_30386_PI426442911
	12	**ubiquitin carboxyl-terminal hydrolase 7-like (*A. florea*)**	0.00E+00	4.35	3.84	4.90	3.78	3.98	CUST_30927_PI426442911
	12	**ubiquitin carboxyl-terminal hydrolase 8 (*X. laevis*)**	5.00E-10	4.78	4.59	5.53	4.81	4.61	CUST_17882_PI426442911
	12	**ubiquitin-conjugating enzyme E2 O (*A. echinatior*)**	3.00E-43	5.72	4.89	5.83	4.90	5.37	CUST_28427_PI426442911
		26S proteasome non-ATPase regulatory subunit 3 (*N. vitripennis*)	0	7.19	8.65	6.39	7.19	7.29	CUST_5946_PI426442911
		26S proteasome non-ATPase regulatory subunit 5 (*B. taurus*)	5.09E-16	4.11	4.57	3.53	4.42	3.41	CUST_17167_PI426442911
		cathepsin B (*L. vannamei*)	2.00E-69	5.04	6.27	4.48	4.61	5.30	CUST_2959_PI426442911
		F-box only protein 21 (*H. sapiens*)	9.14E-12	4.97	5.80	4.84	5.07	4.69	CUST_8580_PI426442911
		kelch repeat-containing protein (*D. discoideum*)	0	12.06	13.31	12.28	12.29	12.18	CUST_6447_PI426442911
		late endosomal/lysosomal Mp1-interacting protein homolog (*T. castaneum*)	9.00E-58	4.62	5.92	4.22	4.51	4.33	CUST_10367_PI426442911
		proteasome alpha 4 subunit (*D. plexippus*)	7.00E-103	7.41	8.23	6.65	7.27	7.41	CUST_7123_PI426442911
		proteasome subunit alpha type-2 (*C. elegans*)	3.07E-81	7.10	8.32	6.55	7.46	7.17	CUST_2259_PI426442911
		proteasome subunit beta type-2 (*C. elegans*)	3.98E-34	7.23	8.20	6.09	7.21	6.80	CUST_5607_PI426442911
		proteasome subunit beta type-5-like (*A. florea*)	2.00E-107	6.93	8.14	6.20	6.87	6.90	CUST_5486_PI426442911
		proteasome zeta subunit (*T. castaneum*)	1.00E-127	6.75	7.67	5.62	6.68	6.36	CUST_7029_PI426442911
…									

Functional category	τ	Annotation[a]	e-value[b]	1:00	6:00	10:00	15:00	18:00	Probe[c]
… **Proteolysis**		RING-box protein 1 (*C. elegans*)	1.57E-48	4.04	4.21	3.24	3.99	3.72	CUST_7268_PI426442911
		similar to CG13322-PA, isoform A (*T. castaneum*)	6.00E-84	4.25	5.06	4.21	3.78	4.14	CUST_8945_PI426442911
		Ubc protein (*P. monodon*)	0	9.52	10.76	9.37	10.04	9.84	CUST_3139_PI426442911
		ubiquitin conjugation factor E4 (*C. elegans*)	6.51E-16	4.42	4.53	3.69	4.33	3.76	CUST_29295_PI426442911
		ubiquitin-conjugating enzyme E2 b (*E. sinensis*)	6.00E-95	10.04	11.32	9.86	10.03	10.35	CUST_5720_PI426442911
		ubiquitin-fold modifier 1 precursor, putative (*A. aegypti*)	2.00E-46	6.19	7.34	5.08	6.29	6.14	CUST_9676_PI426442911
		UBX domain-containing protein (*I. scapularis*)	2.44E-11	3.95	5.07	3.95	4.83	3.50	CUST_19177_PI426442911
		E3 ubiquitin-protein ligase (*X. laevis*)	1.67E-24	6.97	6.58	7.51	7.13	7.11	CUST_15680_PI426442911
**Redox regulation**	24	**selenium-dependent glutathione peroxidase (*M. rosenbergii*)**	7.00E-39	6.97	7.21	5.91	6.44	5.56	CUST_7094_PI426442911
	24	**1-Cys peroxiredoxin (*D. immitis*)**	1.65E-66	7.57	8.47	6.20	7.37	6.90	CUST_3407_PI426442911
	24	**peroxiredoxin 6 (*S. paramamosain*)**	3.00E-124	7.43	8.30	6.27	7.06	6.77	CUST_3408_PI426442911
	24	**selenoprotein T (*C. quadricarinatus*)**	4.00E-36	5.26	6.21	4.97	4.79	4.80	CUST_21619_PI426442911
	12	**ferritin 1 (*E. sinensis*)**	6.62E-23	3.69	4.63	3.04	4.43	4.28	CUST_2459_PI426442911
	12	**hebp2 heme binding protein 2 (*D. rerio*)**	6.00E-29	7.52	8.19	6.17	7.24	7.65	CUST_7360_PI426442911
	12	**ferritin light-chain subunit (*R. exoculata*)**	1.93E-22	3.27	4.51	3.38	4.48	3.95	CUST_2460_PI426442911
	12	**sptzle 2-like protein (*L. salmonis*)**	2.20E-78	4.88	6.07	4.07	4.30	6.92	CUST_398_PI426442911
		ferritin (*L. vannamei*)	0	6.39	7.42	5.45	6.77	6.35	CUST_2645_PI426442911
		ferritin peptide (*F. indicus*)	5.00E-90	9.69	10.53	9.41	9.53	9.26	CUST_7064_PI426442911
		thioredoxin (*E. granulosus*)	9.46E-13	6.99	7.77	5.87	7.20	5.69	CUST_3860_PI426442911
		thioredoxin-like protein (*S. pombe*)	2.97E-38	4.42	5.56	3.55	4.58	3.81	CUST_6023_PI426442911
**Signal transduction**	24	**brefeldin A-inhibited guanine nucleotide-exchange protein 1 (*B. taurus*)**	2.40E-18	5.41	5.28	4.06	4.08	4.32	CUST_26206_PI426442911
	24	**calcium-binding protein p22 (*A. mellifera*)**	7.00E-96	6.53	7.12	5.67	6.16	6.12	CUST_7144_PI426442911
	24	**phosphatidylinositol N-acetylglucosaminyltransferase subunit P (*A. mellifera*)**	4.00E-18	5.36	5.97	4.45	4.72	5.01	CUST_20544_PI426442911
	24	**annexin IX (*D. melanogaster*)**	1.00E-16	2.80	3.53	1.98	2.17	2.21	CUST_30875_PI426442911
	24	**insulin-like growth factor 2 receptor-like (*S. kowalevskii*)**	3.00E-08	5.93	6.04	5.60	5.39	4.93	CUST_21902_PI426442911
	24	**G8 domain-containing protein (*D. discoideum*)**	9.60E-23	2.16	2.17	3.66	3.10	2.79	CUST_1542_PI426442911
	24	**glutamine synthetase (*P. argus*)**	1.06E-35	6.16	5.81	4.68	4.42	5.99	CUST_9886_PI426442911
	12	**glutamate receptor binding protein (*D. melanogaster*)**	9	7.11	6.65	6.50	6.88	6.23	CUST_14400_PI426442911
	12	**glutamate dehydrogenase (*L. vannamei*)**	0	8.98	9.75	7.92	8.70	8.93	CUST_5833_PI426442911
	12	**EsCry (*E. superba*)**	0	5.54	6.58	5.78	6.06	6.06	CUST_32212_PI426442911
…									

Functional category	τ	Annotation[a]	e-value[b]	1:00	6:00	10:00	15:00	18:00	Probe[c]
… **Signal transduction**	12	**protein FAM13A-like (*O. niloticus*)**	4.00E-10	5.76	6.97	5.15	5.92	6.26	CUST_21437_PI426442911
	12	**receptor expression-enhancing protein 2 (*B. taurus*)**	3.11E-28	4.45	4.78	4.98	3.84	4.99	CUST_30829_PI426442911
	12	**arrestin 1 (*D. melanogaster*)**	1.00E-17	4.49	5.69	7.25	4.16	6.01	CUST_16879_PI426442911
	12	**Chitinase-like protein (*S. pombe*)**	6.10E-15	10.18	10.37	11.01	10.41	10.45	CUST_5725_PI426442911
	12	**FERM domain-containing protein 4B (*M. musculus*)**	1.40E-26	4.25	4.40	4.91	4.27	4.19	CUST_23399_PI426442911
	12	**GMP synthase/glutamine amidotransferase protein (*R. etli*)**	1.0E-71	4.99	4.13	6.04	4.15	4.88	CUST_21556_PI426442911
	12	**phospholipase c beta (*C. quinquefasciatus*)**	0.00E+00	7.52	7.42	8.15	7.51	7.42	CUST_21529_PI426442911
		sensor histidine kinase (*B. suis*)	8.96E-46	4.13	1.90	1.95	1.92	1.94	CUST_24515_PI426442911
		arrestin, lateral eye (*L. polyphemus*)	7.31E-54	13.75	14.27	12.83	13.23	12.89	CUST_4249_PI426442911
		compound eye opsin BCRH2 (*H. sanguineus*)	1.85E-138	12.59	14.93	12.87	14.02	12.61	CUST_2149_PI426442911
		histidine triad nucleotide-binding protein 1 (*C. elegans*)	7.30E-37	8.35	8.53	7.47	8.34	7.87	CUST_8976_PI426442911
		hydroxysteroid dehydrogenase-like protein 2 (*X. laevis*)	7.00E-62	5.24	6.12	4.86	5.27	4.99	CUST_4181_PI426442911
		opsin BcRh2 (*H. sanguineus*)	1.00E-159	7.62	9.04	7.33	7.46	6.88	CUST_2152_PI426442911
		phosphatidylinositide phosphatase SAC1 (*S. invicta*)	4.94E-18	4.99	5.57	4.45	5.02	4.31	CUST_22686_PI426442911
		phosphatidylinositol 4-kinase beta (*X. tropicalis*)	1.62E-27	4.15	4.36	4.40	5.30	4.36	CUST_24673_PI426442911
		polehole-like protein (*P. monodon*)	1.00E-31	3.73	2.12	4.11	3.49	4.80	CUST_5828_PI426442911
		PDF receptor (*N. vitripennis*)	7.00E-35	4.76	6.00	4.86	3.99	4.85	CUST_13622_PI426442911
**Stress response and immunity**	24	**DJ-1alpha (*D. melanogaster*)**	5.00E-30	6.75	7.28	6.80	6.52	6.49	CUST_7153_PI426442911
	24	**choline dehydrogenase (*B. suis*)**	2.61E-15	2.54	3.01	3.85	2.96	2.55	CUST_18010_PI426442911
	24	**hemocytin (*B. mori*)**	4.41E-14	2.27	2.71	4.47	4.60	3.24	CUST_29231_PI426442911
	24	**antistasin (*H. discus*)**	4.00E-07	4.66	3.13	3.51	3.07	4.57	CUST_6136_PI426442911
	24	**C-type lectin 1 (*M. japonicus*)**	1.00E-11	5.23	3.97	4.20	3.90	4.68	CUST_25305_PI426442911
	12	**major histocompatibility complex, class II, DR beta 6 (*H. sapiens*)**	0	3.06	2.04	2.01	2.46	2.00	CUST_25274_PI426442911
	12	**heat shock protein 70 (*M. rosenbergii*)**	0	11.92	13.07	11.93	11.94	12.40	CUST_1639_PI426442911
	12	**heat shock protein 90-1 (*P. trituberculatus*)**	0	9.15	10.37	9.10	9.23	9.79	CUST_2306_PI426442911
		alcohol dehydrogenase (*D. rerio*)	0	6.21	7.43	5.52	6.24	6.00	CUST_5429_PI426442911
		alcohol dehydrogenase class-3 (*O. vulgaris*)	2.34E-164	6.01	7.49	5.36	6.30	5.88	CUST_5428_PI426442911
		C-type lectin protein (*F. chinensis*)	2.00E-10	8.54	9.59	7.67	7.93	7.15	CUST_10837_PI426442911
		Glutathione S-transferase 1 (*C. riparius*)	3.32E-27	9.01	9.55	8.35	8.68	8.18	CUST_5661_PI426442911
		Glutathione S-transferase 1(*A. suum*)	4.00E-05	7.89	8.97	7.34	7.47	7.52	CUST_20677_PI426442911
		heat shock 70 (*B. malayi*)	1.16E-31	9.13	10.33	9.09	8.83	9.32	CUST_1646_PI426442911
…									

Functional category	τ	Annotation[a]	e-value[b]	1:00	6:00	10:00	15:00	18:00	Probe[c]
… **Stress response and immunity**		heat shock cognate 70 (*P. monodon*)	0	9.89	11.25	9.83	9.75	10.36	CUST_1641_PI426442911
		heat shock protein 90-1 (*P. trituberculatus*)	0	7.04	8.39	6.26	7.17	7.22	CUST_2300_PI426442911
		mitochondrial aldehyde dehydrogenase 2 (*R. pusillus*)	2.00E-43	5.76	6.88	5.51	6.26	5.40	CUST_3912_PI426442911
		pacifastin (*E. sinensis*)	2.00E-11	5.13	5.21	3.96	4.63	3.27	CUST_11871_PI426442911
		involucrin (*C. griseus*)	8.00E-06	3.84	4.30	5.09	4.47	6.76	CUST_6791_PI426442911
**Structural component of cuticle**	24	**lustrin A (*H. rufescens*)**	2.35E-22	6.11	6.82	5.29	5.69	5.19	CUST_5904_PI426442911
	12	**cuticle protein AMP1A (*H. americanus*)**	7.82E-23	4.51	5.71	4.89	4.58	6.44	CUST_221_PI426442911
	12	**putative transglutaminase (*D. plexippus*)**	4.00E-15	8.78	9.73	9.15	7.79	9.80	CUST_12193_PI426442911
	12	**crustacyanin-C1 subunit (*H. gammarus*)**	3.51E-42	5.09	4.20	5.64	4.44	4.90	CUST_8000_PI426442911
		arthrodial cuticle protein AMP16.3 (*C. sapidus*)	9.00E-27	12.05	13.15	11.75	12.11	11.89	CUST_4731_PI426442911
		arthrodial cuticle protein AMP16.3 (*C. sapidus*)	1.00E-27	10.51	11.99	10.39	10.50	9.88	CUST_8830_PI426442911
		cuticle proprotein proCP5.2 (*C. sapidus*)	4.94E-39	8.10	8.22	9.59	8.36	9.90	CUST_892_PI426442911
**Transcription**	24	**exosome complex exonuclease RRP43 (*B. taurus*)**	2.69E-16	4.75	4.73	4.56	4.18	3.88	CUST_10671_PI426442911
	24	**putative small nuclear ribonucleoprotein, core (*D. plexippus*)**	9.00E-21	4.01	5.66	4.71	4.86	4.28	CUST_17322_PI426442911
	24	**U2 small nuclear ribonucleoprotein A (*M. musculus*)**	1.59E-12	4.73	4.98	5.26	4.91	4.74	CUST_31566_PI426442911
	12	**polycomb protein PCL_c (*D. melanogaster*)**	7.17E-46	7.72	6.95	7.02	7.16	6.95	CUST_5459_PI426442911
	12	**zinc finger protein 22 (*H. sapiens*)**	6.00E-08	7.89	7.13	7.19	7.58	6.96	CUST_16664_PI426442911
	12	**transcriptional repressor p66-beta (*M. musculus*)**	6.13E-11	7.99	8.13	7.86	8.35	8.36	CUST_9548_PI426442911
	12	**peregrin (*H. sapiens*)**	1.42E-17	5.97	6.38	6.54	6.23	6.72	CUST_27486_PI426442911
	12	**chromatin-remodeling complex ATPase chain isw-1 (*C. elegans*)**	6.29E-48	5.71	5.63	6.02	5.41	5.97	CUST_29900_PI426442911
	12	**transcriptional repressor NF-X1 (*C. elegans*)**	1.93E-17	3.23	3.54	4.44	3.38	3.58	CUST_25309_PI426442911
	12	**zinc finger protein 2 homolog (*H. sapiens*)**	7.12E-33	2.67	2.73	4.44	3.00	3.46	CUST_27200_PI426442911
	12	**GTP-binding protein 128up-like isoform 1 (*A. mellifera*)**	0	3.87	3.49	4.72	3.87	4.23	CUST_5930_PI426442911
	12	**THO complex, putative (*A. aegypti*)**	6.0E-18	3.35	2.94	4.46	3.05	3.64	CUST_11304_PI426442911
	12	**splicing factor, arginine/serine-rich 6 (*S. salar*)**	1.66E-12	7.61	7.27	8.03	7.71	7.48	CUST_10719_PI426442911
	12	**zinc finger protein 420 (*B. taurus*)**	5.49E-12	6.64	6.08	7.04	6.20	6.01	CUST_4612_PI426442911
	12	**serine/arginine-rich splicing factor 6 (*B. taurus*)**	9.13E-13	8.15	7.47	8.38	8.28	7.91	CUST_8874_PI426442911
	12	**transcriptional repressor rcnR (*S. choleraesuis*)**	2.33E-11	7.52	5.60	7.21	6.60	6.34	CUST_15809_PI426442911
		ATP-dependent RNA helicase ddx54 (*D. discoideum*)	3.08E-12	4.08	2.46	2.63	2.50	2.37	CUST_10653_PI426442911
		eukaryotic initiation factor 4A (*C. sapidus*)	0	7.23	8.08	6.36	6.86	7.28	CUST_75_PI426442911
…									

Functional category	τ	Annotation[a]	e-value[b]	1:00	6:00	10:00	15:00	18:00	Probe[c]
… **Transcription**		heterogeneous nuclear ribonucleoprotein A3 homolog 2 (*X. laevis*)	1.08E-19	12.00	12.77	11.39	12.07	11.73	CUST_1529_PI426442911
		RNA-binding protein 8A TSUNAGI (*A. mellifera*)	4.00E-54	5.60	6.40	4.16	4.94	4.89	CUST_5492_PI426442911
		similar to lupus la ribonucleoprotein (*T. castaneum*)	2.00E-61	5.36	6.53	4.86	5.72	5.52	CUST_5994_PI426442911
		similar to poly A binding protein (*T. castaneum*)	0	9.33	9.84	8.95	8.96	9.16	CUST_9_PI426442911
		chromodomain-helicase-DNA-binding protein Mi-2 homolog (*D. melanogaster*)	9.02E-78	9.15	9.01	9.56	9.20	9.39	CUST_6743_PI426442911
		cleavage stimulation factor subunit 1 (*A. suum*)	3.00E-38	4.27	3.19	5.01	3.73	4.71	CUST_28442_PI426442911
		PR domain zinc finger protein 9 (*H. sapiens*)	2.00E-12	5.36	4.74	5.58	5.25	5.53	CUST_4843_PI426442911
		protein furry (*D. melanogaster*)	6.48E-41	5.73	5.45	6.39	6.01	5.69	CUST_14305_PI426442911
		pumilio-like protein 1 (*H. saltator*)	3.16E-17	8.76	8.67	9.13	8.74	9.02	CUST_9584_PI426442911
		heterogeneous nuclear ribonucleoprotein U-like protein 1 (*H. sapiens*)	3.59E-35	8.61	8.82	8.37	9.05	8.45	CUST_14907_PI426442911
		heterogeneous nuclear ribonucleoprotein U-like protein 1 (*H. sapiens*)	1.52E-29	6.82	7.42	6.92	7.46	6.94	CUST_16768_PI426442911
		histone-lysine N-methyltransferase pr-set7 (*D. melanogaster*)	1.17E-21	2.28	3.47	2.91	4.24	3.22	CUST_22836_PI426442911
		protein sex-lethal (*H. saltator*)	2.22E-12	2.45	2.20	2.43	3.38	2.58	CUST_20763_PI426442911
		staufen-like protein 2 (*C. floridanus*)	3.00E-38	8.92	8.60	9.19	8.82	9.31	CUST_22911_PI426442911
		trithorax group protein osa (*D. melanogaster*)	5.97E-16	5.27	5.30	5.76	5.34	6.08	CUST_12584_PI426442911
		zinc finger protein 624 (*H. sapiens*)	1.16E-12	2.90	3.81	3.89	3.78	4.29	CUST_16024_PI426442911
**Transport**	24	**BET1 homolog (*R. norvegicus*)**	4.36E-16	5.80	5.46	3.81	4.51	3.46	CUST_8006_PI426442911
	24	**prenylated Rab acceptor protein 1 (*S. scrofa*)**	1.95E-13	4.55	4.58	3.17	3.47	3.15	CUST_13195_PI426442911
	24	**copa; coatomer protein complex, subunit alpha (*D. rerio*)**	9.00E-27	4.43	4.86	4.12	3.96	3.81	CUST_14207_PI426442911
	24	**synaptoporin (*R. norvegicus*)**	2.73E-12	4.98	5.26	4.21	4.56	3.98	CUST_7505_PI426442911
	24	**solute carrier family 35 member B1 homolog (*A. pisum*)**	1.00E-55	4.57	5.29	4.24	3.62	4.15	CUST_4639_PI426442911
	24	**excitatory amino acid transporter (*O. volvulus*)**	8.08E-13	8.99	9.43	9.08	8.37	8.59	CUST_13105_PI426442911
	24	**WSDV receptor Rab7 (*M. japonicus*)**	7.00E-26	4.68	5.73	4.64	4.42	4.18	CUST_16163_PI426442911
	24	**sodium-dependent glutamate/aspartate transporter 2 (*M. fascicularis*)**	3.00E-20	8.59	9.64	8.96	8.41	8.52	CUST_23963_PI426442911
	24	**solute carrier organic anion transporter family member 5A1-like (*A. florea*)**	2.00E-26	13.72	13.08	14.68	14.58	14.97	CUST_11360_PI426442911
	12	**Ran-binding protein 3-like (*M. musculus*)**	5.70E-22	3.79	3.51	3.29	3.69	3.00	CUST_27883_PI426442911
	12	**hippocampus abundant transcript-like protein 1 (*B. taurus*)**	1.61E-34	5.71	6.32	5.04	6.08	5.60	CUST_15521_PI426442911
	12	**AP-1 complex subunit sigma-2 (*D. discoideum*)**	2.94E-44	5.30	6.67	5.01	5.63	6.14	CUST_3958_PI426442911
	12	**ATPase, P-type (transporting), HAD superfamily, subfamily IC (*H. denitrificans*)**	3.00E-12	3.01	2.75	3.52	2.35	3.39	CUST_30434_PI426442911
	12	**deoxyribonuclease tatD (*H. saltator*)**	1.77E-24	4.50	4.86	5.12	3.94	5.07	CUST_8613_PI426442911
	12	**myosin-Vb (*R. norvegicus*)**	9.69E-12	4.18	2.85	4.61	3.83	2.98	CUST_13615_PI426442911
…									

Functional category	τ	Annotation[a]	e-value[b]	1:00	6:00	10:00	15:00	18:00	Probe[c]
... **Transport**	12	**solute carrier family 35 member F5 (*B. taurus*)**	3.88E-31	5.05	4.38	5.36	4.51	4.57	CUST_20635_PI426442911
		Bestrophin-2 (*H. saltator*)	2.0E-36	6.51	5.72	6.06	5.24	5.71	CUST_23791_PI426442911
		ADP-ribosylation factor 1 (*B. malayi*)	5.00E-31	10.10	10.85	10.11	10.12	10.06	CUST_5375_PI426442911
		ADP-ribosylation factor 1 (*L. migratoria*)	2.37E-84	7.09	7.73	6.79	6.94	6.63	CUST_6342_PI426442911
		ADP-ribosylation factor GTPase-activating protein (*S. paramamosain*)	5.00E-05	5.98	6.10	5.82	6.04	5.12	CUST_31369_PI426442911
		Arf gtpase-activating protein (*A. aegypti*)	8.57E-14	5.04	5.92	4.30	5.20	4.57	CUST_9345_PI426442911
		coatomer subunit alpha (*D. discoideum*)	2.11E-46	8.10	8.96	7.03	7.83	7.51	CUST_7066_PI426442911
		GTP-binding protein sar1 (*D. plexippus*)	1.00E-34	7.92	8.59	7.12	7.47	7.48	CUST_5415_PI426442911
		niemann-Pick type C-related protein 1 (*S. cerevisiae*)	2.40E-12	5.46	6.06	4.54	5.29	4.75	CUST_22069_PI426442911
		oligosaccharyltransferase complex subunit ostc (*X. tropicalis*)	9.13E-59	6.75	7.78	6.86	7.30	6.25	CUST_5604_PI426442911
		outer mitochondrial membrane complex subunit TOM22 translocase (*A. variegatum*)	1.00E-14	5.80	6.66	4.80	5.38	5.84	CUST_6104_PI426442911
		Rab gdp-dissociation inhibitor (*T. castaneum*)	0	9.13	10.34	8.94	8.94	9.17	CUST_5709_PI426442911
		Rab-protein 11 (*D. melanogaster*)	2.00E-106	7.61	8.52	7.48	7.74	7.68	CUST_5378_PI426442911
		Sec61 alpha 1 subunit (*A. pisum*)	0	9.66	10.30	9.40	9.55	9.71	CUST_5807_PI426442911
		sodium potassium-transporting ATPase subunit beta (*L. vannamei*)	2.00E-142	7.53	8.49	6.90	7.30	7.50	CUST_2781_PI426442911
		surfeit 4 cargo protein (*A. aegypti*)	3.00E-118	6.10	6.81	5.55	6.08	5.91	CUST_5869_PI426442911
		trafficking protein particle complex 5 (*S. paramamosain*)	2.00E-45	5.97	7.20	5.52	5.35	5.52	CUST_9143_PI426442911
		transport protein Sec23A (*X. tropicalis*)	1.00E-15	8.37	9.17	7.66	8.13	7.78	CUST_20356_PI426442911
		V-type proton ATPase subunit E (*D. melanogaster*)	2.00E-91	4.99	6.35	4.21	5.26	5.17	CUST_5420_PI426442911
		Y+L amino acid transporter 2 (*H. saltator*)	6.67E-22	6.24	6.48	5.78	6.30	5.87	CUST_12241_PI426442911
		ABC transporter G family member 23 (*D. discoideum*)	2.77E-32	2.74	2.68	4.66	3.42	3.62	CUST_15701_PI426442911
		guanine nucleotide exchange factor (*A. aegypti*)	4.00E-69	9.63	9.30	10.16	9.38	9.97	CUST_9453_PI426442911
		L-arabinose transport system permease protein AraH (*S. flexneri*)	6.52E-34	2.81	2.51	4.88	3.88	3.22	CUST_23729_PI426442911
		golgi phosphoprotein 3-like A (*X. laevis*)	3.86E-23	3.89	4.49	3.68	5.08	4.02	CUST_16128_PI426442911
		calcium-activated potassium channel slo-1 (*C. elegans*)	2.21E-27	6.74	5.89	6.98	6.78	6.99	CUST_13950_PI426442911
		exportin-2 (*D. melanogaster*)	5.41E-13	3.52	3.34	3.99	3.28	4.34	CUST_25956_PI426442911
		mex-3 protein (*T. castaneum*)	2.00E-29	5.15	5.16	5.40	5.07	5.86	CUST_13372_PI426442911
