# Supplementary material for: The Antarctic Krill Euphausia superba Shows Diurnal Cycles of Transcription under Natural Conditions
Source: PLoS One. 2013 Jul 17;8(7):e68652. doi: 10.1371/journal.pone.0068652 (PMC3714250; doi:10.1371/journal.pone.0068652)
Supplement: Table S2 — Primers used in quantitative RT-PCR. (PDF) [file pone.0068652.s006.pdf]

Table S2. Primers used in quantitative RT-PCR.

# Isotig/singleton	Description	Forward Primer (5'-3')	Reverse Primer (5'-3')
**isotig06063**	26S proteasome non-ATPase regulatory subunit 3	CCTAGAAAGTGCTGAGGAACG	ATCCTCCTCTGCCATCTCTTT
**FW09YNP01ATTT0**	arrestin 1	TGCAAGGAATAGCTGTAAACTGTC	TGGTTTCTTGCTTTCACTCAGA
**isotig03868**	citrate synthase 2	ACCTCTTGAACGACCAAAGTCT	CCAGCCTTAGCCAGTTTAGC
**isotig01544**	heat shock cognate 70	CCTGGGAGGTGAAGACTTTG	TCCTTCTTGTACTTACGCTTGAAC
**isotig02214**	heat shock protein 90-1	TGCTGGTGGCTCTTTCACTA	AGATGCAGGACGATCTTGGT
**isotig02063**	opsin BcRh2	CGAACCGGAACACTTTCATT	TGGATCCATGCCAAAGAAT
**isotig03346**	Peroxiredoxin 6	CAGATGTTCCCTCAGCATAAAGT	GCTGAGGTGTTGTACGCAAA
**FW09YNP01EWHJ7**	Phosphatidylinositide phosphatase SAC1	TACAGCCGGAAACGAGTCTT	CAATGCTTGCTTGTTTAATGGA
**isotig05837**	ubiquitin-conjugating enzyme E2	TCAATAAATGGAAATACTCGTGGA	GCCAATCATGTACCCTTATGAAT
